# Electro‐Thermally Controlled Active Mechanical Metamaterials with Programmable Stiffness and Nonreciprocity

**DOI:** 10.1002/advs.202511669

**Published:** 2025-08-28

**Authors:** Jai Dunne, Robert D. Crapnell, Krzysztof K. Dudek, Tom Allen, Craig E. Banks, Olly Duncan

**Affiliations:** ^1^ Faculty of Science and Engineering, Dalton Building Manchester Metropolitan University Manchester M1 5GD UK; ^2^ Institute of Physics University of Zielona Gora ul. Szafrana 4a Zielona Gora 65‐069 Poland

**Keywords:** active metamaterials, 4D printing, smart materials

## Abstract

Active mechanical metamaterials have the potential to revolutionize material capabilities, by switching between different properties. The active mechanical metamaterial presented here can be remotely programmed to switch between compressive and shear deformation modes that cause stark changes in stiffness. The considered metamaterial uses controlled instabilities to change the buckling mode of electro‐thermally activated beams. The beams form electrical circuits. When selectively charged, they heat (and soften). The effects of manufacturing imperfections are overcome by connecting the beams to a compliant mechanism, allowing reliable control over the compressive buckling modes that cause the stiffness changes. Connection points in the metamaterial resemble a fish‐bone structure, known to exhibit static nonreciprocity, which is actively controllable within the considered metamaterial. As such, it is shown (computationally) that this metamaterial is capable of modulating traction and pressure across a surface. Pressure can be doubled between adjacent unit‐cells while traction can be shielded (i.e., zero) in selected regions. This concept has potential applications in robotic gripper interfaces, and medical devices.

## Graphical abstract

Actively programmable mechanical metamaterial, featuring smart conductive polymers, designed instabilities, and compliant mechanisms. Capable of stiffness switching, local surface pressure modulation, and nonreciprocal shear relationships that allow local shielding from surface traction. Potential applications in soft robotic grippers and smart medical devices.

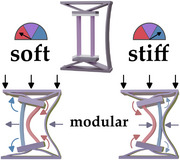

## 1 Introduction

Embedding smart materials into mechanical metamaterials provides exciting opportunities to actively programme and control a broad range of material properties.^[^ ^1^, ^2^, ^3^, ^4^, ^5^, ^6^, ^7^ ^]^ The core concept of mechanical metamaterials is the design of material structures to have unusual or counterintuitive properties.^[^ ^8^, ^9^, ^10^, ^11^, ^12^, ^13^, ^14^, ^15^, ^16^, ^17^, ^18^, ^19^ ^]^ As such, mechanical metamaterials have widened our available range of useful material properties. Smart materials, on the other hand, change their properties according to external stimuli, allowing controlled modulation of material responses.^[^ ^20^, ^21^, ^22^, ^23^, ^24^, ^25^, ^26^ ^]^ Combining these concepts, smart materials can be used to control and tune the vast array of properties that mechanical metamaterials can achieve.^[^ ^13^, ^27^, ^28^, ^29^, ^30^ ^]^

Some of the properties that can be achieved using mechanical metamaterials include negative Poisson's ratio,^[^ ^14^, ^31^, ^32^, ^33^, ^34^, ^35^ ^]^ negative stiffness,^[^ ^19^, ^36^ ^]^ and nonreciprocity.^[^ ^2^, ^3^, ^4^, ^5^, ^8^ ^]^ While negative Poisson's ratio and negative stiffness are self‐descriptive terms, nonreciprocity relates to signal transmission between two opposing points on a body. When a signal (e.g., applied load) is mirrored, convention suggests that the response (e.g., deformation of the opposing point) should also be mirrored.^[^ ^37^ ^]^ Using material nonlinearities or structural instabilities, it is possible to design mechanical metamaterials that break this fundamental relationship.^[^ ^2^, ^3^, ^4^, ^5^, ^8^ ^]^

Many of the counterintuitive properties for which mechanical metamaterials are known provide benefits in applications where standard material properties limit functionality. For example, negative stiffness can increase damping,^[^ ^38^ ^]^ while auxetic materials can have high indentation resistance.^[^ ^39^ ^]^ Nonreciprocal mechanical metamaterials allow passive changes between loading surface, providing the potential to mitigate loads from impacts on a protective interface without affecting those applied by the person or object being protected.^[^ ^40^ ^]^ Control over such properties shows promise within high‐value, rapid‐growth markets, such as robotics and medical devices.

Various mechanisms have been used to actuate active mechanical metamaterials, including magnetic fields,^[^ ^41^, ^42^, ^43^, ^44^, ^45^ ^]^ humidity,^[^ ^27^, ^46^ ^]^ temperature,^[^ ^47^, ^48^, ^49^ ^]^ light,^[^ ^50^, ^51^, ^52^ ^]^ and micro‐actuators/motors.^[^ ^53^ ^]^ The specific mechanisms that allow thermal actuation include the use of polymer thermal response,^[^ ^48^, ^49^, ^54^ ^]^ inclusion of heat sensitive solid^[^ ^55^ ^]^ or liquid^[^ ^56^ ^]^ metals, and embedded circuitry such as highly resistive wire heating elements.^[^ ^57^ ^]^ Conductive polymers have also been applied to mechanical metamaterials as a mechanism to sense deformation modes.^[^ ^58^ ^]^ Given that many of these active metamaterials are produced by additive manufacturing, and change their structure over time, they are often referred to as 4D printed metamaterials.^[^ ^47^ ^]^ These active mechanical metamaterials do, however, remain costly and labor intensive to develop. This is because their design couples multiple types of physics, and they are typically fabricated from many intricate components.

The effect of the actuation mechanisms in active mechanical metamaterials is often amplified, either by designing them to act on a compliant mechanism,^[^ ^27^, ^42^, ^50^, ^51^ ^]^ typically used for shape morphing, or to affect a structural instability to amplify response changes.^[^ ^3^, ^30^, ^40^ ^]^ For example, a slender beam made from two materials (side‐by‐side) of different stiffness (i.e., a bi‐beam) should buckle toward its stiffer side when compressed. By changing which side of the bi‐beam is stiffer, buckling direction can be controlled. This has been demonstrated in passively adaptive mechanical metamaterials, whereby material rate dependence switches the order of beam stiffness to control buckling direction, and whether self‐contact between pairs of beams will occur.^[^ ^30^ ^]^ Contact between buckling beams generally causes overall stiffening,^[^ ^59^ ^]^ and changes in response that may appear as deformation increases,^[^ ^30^, ^60^, ^61^ ^]^ or in subsequent loading cycles.^[^ ^62^, ^63^, ^64^ ^]^

Structural instabilities such as buckling can, however, be sensitive to manufacturing imperfections and loading conditions.^[^ ^65^ ^]^ To realize the vast potential of active mechanical metamaterials, designs that can be reliably actuated despite imperfections are needed. Addressing such cost and scale‐up design challenges is key to increasing the transfer of active mechanical metamaterials from low technology readiness level research to wider application.

Conductive polymers can be additively manufactured as electrochemical sensors.^[^ ^58^, ^66^, ^67^, ^68^ ^]^ This means that there is potential for the heat generated from their electrical resistance to be used to actively soften them. The various polymers used in conductive filaments include Polylactic Acid (PLA), Acrylonitrile Butadiene Styrene, Polypropylene (PP), and Thermoplastic Polyurethane (TPU).^[^ ^67^, ^69^, ^70^, ^71^ ^]^ Conductive additives include Carbon Black, graphite, and Graphene.^[^ ^67^, ^69^, ^71^ ^]^

While these conductive polymers appear promising for application in active mechanical metamaterials, they have been developed primarily for electrochemical sensing. This means that their mechanical functionality is rarely considered.^[^ ^66^, ^67^ ^]^ Low bulk resistance improves electrochemical sensing, and this is typically achieved by increasing the amount of additive.^[^ ^69^ ^]^ This high additive content limits their flexibility. Based on their low bulk resistance, however, it is unclear whether these filaments would heat, and hence soften, under an electrical charge. Conversely, because these filaments have already been applied as low‐cost sensors,^[^ ^72^, ^73^ ^]^ their use in active mechanical metamaterials has a clear route from prototyping (via. additive manufacturing) to scale‐up (via. molding), and may provide multifunctionality such as concurrent sensing and adapting.

Given that these conductive polymers have potential to provide a scalable platform technology to develop active and programmable metamaterials, with sensing capabilities,^[^ ^66^, ^67^ ^]^ we explore how they can be designed and used to control mechanical responses. To do this, we harness a combination of compliant mechanisms and structural instabilities, aiming for stark, reliable switches in stiffness, alongside programmable nonreciprocity. A potential application is demonstrated; fine control over local pressure, traction, and dexterity for the interface of a robotic gripper, or medical device.

## 2 Concept Development

**Figure 1:**
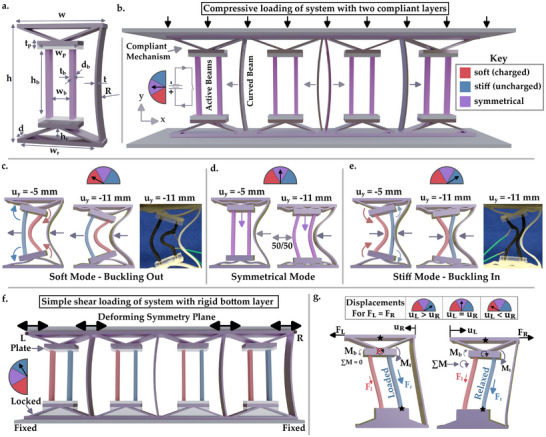
a) Schematic showing the dimensions of a unit‐cell. Heights are denoted *h*, widths are *w*, depths are *d*, and thicknesses are *t*. Subscripts denote whether the dimension relates to a beam (b), compliant mechanism/rocker (r), or plate (p). All flexing sections in the surrounding structure also have thickness t, and only the beams have the reduced depth of *d*_*b*_. b) The mechanical metamaterial under compression. c–e) Schematics, and images from single unit‐cell tests, of the programmable deformation modes following compression (u) of 5 and 11 mm: c) Soft mode, whereby the inner beams consisting of smart polymers are softened (red) ‐ resulting in early bucking and delayed internal contacts. d) Symmetrical mode, whereby both beams are either soft or stiff, causing delayed buckling and a random buckling direction. e) Stiff mode, whereby the outer beams are softened, resulting in a stiff response dominated by early self‐contact. f) A variation of the metamaterial and loading conditions, with a layer of compliant mechanisms locked allowing programmable static nonreciprocity. g) The nonreciprocal deformations. Stars show effective connections. Notation shows moments (M) and forces (F) from left (subscript L) and right (subscript R) beams acting on the plates as a result of differences in tensile load (subscript t), and bending moments (subscript b). These either cancel or cause rotation of the compliant mechanism that respectively increase or relax the tensile load on the stiffer beam. All applied loads are shown using black arrows.

The proposed active mechanical metamaterial takes inspiration from passively adaptive bi‐beams with programmable stiffness,^[^ ^30^ ^]^ and fish‐bone inspired structures that show static nonreciprocity.^[^ ^74^ ^]^ Our concept uses three components: i) Two separated, active beams, shown as purple in **Figure** **1**a,b, that can be selectively softened. ii) A compliant mechanism that the two curved beams connect to. This mechanism consists of a flat plate that the active beams connect to, and two diagonal beams that provide compliance. iii) The relatively stiff, inactive top and bottom plates that the compliant mechanism connects to are connected with a curved beam on one side of each unit‐cell.

Considering the compressive response, structural instabilities that affect compressive bucking direction and (therefore) self‐contact between geometric features are used to amplify the effect of beam softening. The two active beams and the connected compliant mechanism deform concurrently, while the compliant mechanisms rotate under a relatively small torque. This mechanism amplifies the effect of the imbalance induced by selectively softening one of the active beams (electro‐thermally). This rotation induced on the compliant mechanism allows the buckling direction of the active beams to be reliably controlled, by amplifying their end conditions, to mitigate manufacturing or loading imperfections (Figure 1c,e). The curved beam placed on one side of this mechanism is designed to always flex in the same direction when the metamaterial is compressed. This means it will either contact the active beams, or not, depending on their controlled buckling direction. Each pair of active beams and compliant mechanisms, along with one curved beam, form a unit‐cell (Figure 1c–e). The unit‐cells have stiff couplings, which prevent transmitted tractions and moments under a uniaxial load, so emergent effects are not expected ‐ simplifying the design (see Section S2, Supporting Information).

A different active beam stiffness on each side of the compliant mechanism causes the softer (charged) beam to compress by more than the stiffer (uncharged) one. This can cause the compliant mechanisms to rotate, which in turn controls the buckling direction (Figure 1c–e). The direction of buckling can occur away from (Figure 1c), or toward (Figure 1e), other structural features (in this case the curved beam, Figure 1c). When the active beams buckle toward the curved beam and make contact, the metamaterial has a stiffer response. As such, selective softening of an active beam switches the metamaterial between a soft mode (Figure 1c) and a stiff mode (Figure 1e). A third (symmetrical) mode is also available whereby both active beams have equal stiffness (i.e., both are uncharged or equally charged, Figure 1d). This symmetry prevents the mechanism from rotating, delaying the onset of buckling, and randomizing its direction.

The active beams were additively manufactured from conductive polymers,^[^ ^69^ ^]^ and their softening controlled by direct circuitry. Charging an active beam (with a potential difference of 20 V) heats (to ∼110°C) and softens it (by a factor of approximately five). The compliant mechanism tilts toward the stiffer beam, meaning that buckling of the active beams will also occur in that direction. The proposed design is robust, because: i) The beams are separated, amplifying the relative moment that switches their buckling direction. ii) The relative moment between beams is further amplified by a rotating compliant mechanism. iii) Self‐contact happens between switchable components and a passive, curved beam (halving the probability of an active member of a contacting pair buckling in the unintended direction).

The key design parameters for the system activation are: i) The stiffness of the compliant mechanism (relating to the diagonal rib thickness). ii) The stiffness (i.e., thickness) of, and distance between, the active beams. iii) The stiffness (i.e., thickness) of the curved beam, and separation between the active and the curved beams. The softening of each active beam should allow its compressive stiffness to switch to become lower than that of the compliant mechanism, so that a rotation of the connective plate can be induced. Their separation should be such that the induced moment causes rotation of the plate when a beam is softened. If their separation is too small, the induced moment may be too small. If their separation is too large, the plate rotation would require a substantial amount of deformation of the softened beam to achieve a sufficient slope to influence the buckling direction. The function of the curved beam is to constrict the flexure space available for the active beams, as required. To ensure that the contribution to compressive stiffness before this point is minimal, the curved beam should generally have a lower stiffness than the active beams (in this case half ‐ see Section S2, Supporting Information). The separation between the active beams and the curved ones changes the point at which the self‐contact, and secondary stiffening, occurs.

Another advantage of separating the beams is seen when one row of compliant mechanisms is locked, by inserting rigid blocks, before transverse deformations are applied (Figure 1f, g). When one active beam is softened, the point of contact at the end of the other one (i.e., where it meets the plate), and the center of connection of the compliant mechanism (i.e., the center of the unit‐cell), are not vertically aligned (see marked stars in Figure 1g). By inserting rigid blocks to lock out one layer of the compliant mechanisms (e.g., the bottom layer), and softening (charging) one beam, we create a diagonal between the stiffer (uncharged) beam and the stiff top or bottom plate. As the deformation is dominated by the stiffer beam, this effectively resembles half of a fish‐bone structure, known to cause static‐nonreciprocity,^[^ ^74^ ^]^ which can be fully represented by applying a symmetry plane (Figure 1f).

Applying the transverse deformation to the central surface of this fishbone‐like structure will mean the beams cause moments and direct tensile loads on the compliant mechanism (Figure 1f,g). The tensile loads will be higher on the side of the stiffer (uncharged) beam, irrespective of the direction of the applied load, meaning that the resultant moment is unchanged when deformation occurs to the left‐ or right‐hand side. Conversely, the bending moments at the end of the flexing beam and the point of attachment to the compliant mechanism will reverse their direction when the loading direction is changed. As such, the sum of moments acting on the compliant mechanism will change with loading direction. When loading is aligned to the side with the softened (charged) beams, the two moments acting on the compliant mechanism will be opposing. When loading is aligned away from the side with the softened beams, the two moments will act in the same direction, rotating the connective plate. This rotation of the compliant mechanism relaxes the stiffer beam, causing a programmable change in the relationship between i) a load applied at point L and a deformation applied at point R, or ii) a load applied at point R and a deformation applied at point L (Figure 1f,g). This is programmable static nonreciprocity.^[^ ^1^, ^2^, ^3^, ^4^, ^5^, ^8^ ^]^ The features that contribute to the nonreciprocity are torsional stiffness of the compliant mechanism, the separation between the active beams, and their end conditions. The curved beam has a low shear stiffness, and so causes minimal contribution (see Section S2, Supporting Information).

## 3 Results and Discussion

### 3.1 Programmable Stiffness

**Figure 2:**
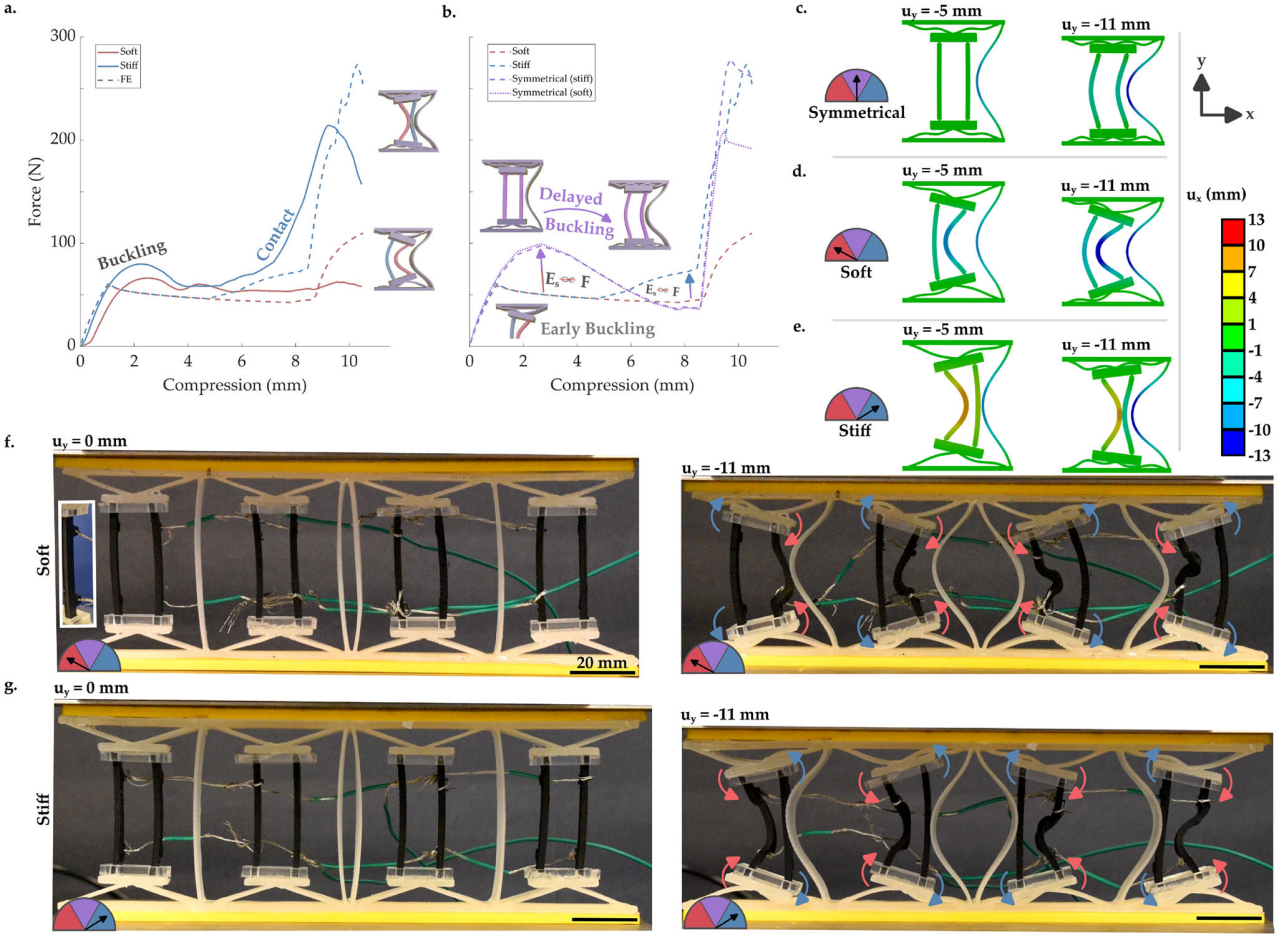
a) Force vs. displacement data for the two active states (both finite element (FE) simulations, and experimental data). Schematics show which beams are selectively softened and the resulting bucking direction. b) Force vs. displacement data (from FE simulations), also including the symmetrical system (with softened beams, and uncharged stiff beams). c–e) False color plots showing x deformation at 5 and 11 mm of compression, for circuits producing the c) symmetrical mode, d) soft mode, and e) stiff modes. f–g) images at 0 and 11 mm compression for the circuits causing a f) soft and g) stiff mode (with all scale bars being 20 mm). Insert in f), (*u*_*y*_ = 0 mm) shows loops used to connect the circuits. The false color plot on the right hand side applies to c–e), while the x‐y axes apply to all.

In this section, the programmed modes in (Figure 1c–e) are demonstrated, by connecting the required circuits and applying 12 mm (20%) compression to the metamaterial, quasistatically (**Figure** **2**). Both experiments and simulations show that buckling occurred at about 2 mm (∼3%) of compression for both structures with selectively softened beams (Figure 2). A clear change in stiffness after buckling at about 8 mm (∼12%) of compression was seen, particularly for the stiff mode. The transitions are more abrupt in the simulation data, which has idealized geometries, material models, and applied boundary conditions (free from the imperfections that influence the abruptness of buckling and self‐contact effects). The stiff mode exhibited about three times higher force than the soft one at 10 mm (∼17%) of compression (Figure 2a). This overall change in stiffness exceeds that of the beam material softening (from charging) alone. In the center of a unit‐cell, the cross‐sectional area of the softened beam was a third of the area of the three beams located there (i.e., the active pair and the curved beam). The beam softening factor of five, divided by the relative area of three, is approximately half of the total softening factor.

During the symmetrical response (whereby both active beams have similar stiffness) buckling was delayed, because the compliant mechanisms did not rotate (Figure 2b,c). The resulting stiffness was not affected by that of the active beams, provided that they were both equal; the response was dominated by the compliant mechanism. This meant that, if we consider the mechanism with two softened beams (i.e., symmetrical (soft) in Figure 2b), stiffening one of them effectively reduced the stiffness (i.e., soft in Figure 2a,b). This was because the stiffer beam caused the compliant mechanism to rotate, switching the beams from relatively stiff compressive deformation to (more compliant) buckling (Figure 2b,d).

As well as causing a switch between free deformation and self‐contact, the stiff mode causes the stiffer (uncharged) active beam to become trapped between the softened active beam and the curved one, providing additional support (Figure 2e). Conversely, the soft mode softens the (charged) active beam that is closer to the curved beam, meaning that if there is some unintended contact between these as a result of fabrication or loading imperfections, the response change would still be partially maintained. As such, while in the experiment one softened beam did buckle in the unintended direction, causing self‐contact (Figure 2f, right image, left‐hand unit‐cell), the effect was mitigated.

Buckling of the softened beam that caused the unintended self‐contact (Figure 2f) was initiated close to the point where the circuit was connected. Inspecting the outputs from the thermal imaging camera (Figure 5a; Section S4, Supporting Information), this connection point maybe be softer than other locations (as the equilibrium temperature is marginally higher, and this temperature is reached sooner). As such, the effect of manufacturing imperfections may be amplified here. In this case, this caused the erroneous buckling. Nonetheless, all eight compliant mechanisms rotated in the intended direction during both experiments, despite this beam buckling in the unintended direction (Figure 2f,g). This meant that the unintended self‐contact occurred between a soft beam with end loads acting away from the unintended self‐contact. As such, this approach of using instabilities such as buckling in combination with compliant mechanisms shows promise to filter out the effects of manufacturing imperfections and unpredictable loading conditions. For further analysis of emergent effects when varying the number of unit‐cells, and variable beam stiffness, the reader is referred to the Section S2 (Supporting Information).

**Figure 3:**
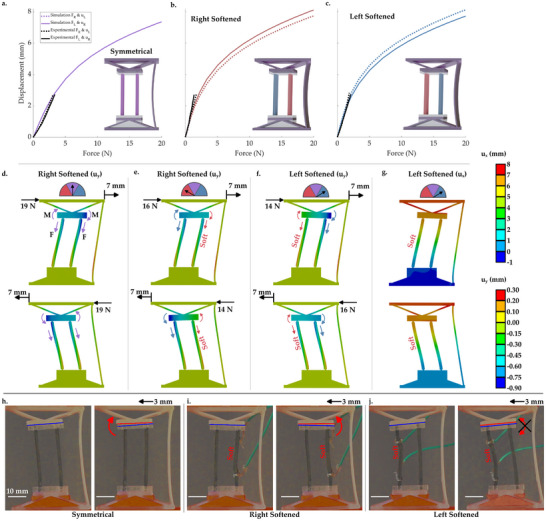
a–c) Displacement magnitude vs. force data for the system with programmable nonreciprocity under simple shear loading, for a) symmetrical beams, b) right beam softened, c) left beam softened. d–f) FE false color plots showing the magnitude of y‐axis deformation, with annotations showing the direction and relative magnitude of tensile force and moment applied by respective stretching and flexing of the ribs, for d) symmetrical beams, e) beams on the right hand side softened, and f) beams on the left side softened. g) False color plot of x deformation (left side beams softened). Top images show 7 mm rightward deformation and the resulting force, bottom images show 7 mm leftward deformation and the resulting force. h–i) Images of tests (with leftward deformation) showing h) the symmetrical system, i) the right beam being softened, j) the left beam being softened, with annotations showing rotation of the compliant mechanism. Left images show an undeformed unit‐cell, right images show a deformed one. For a–c), the simulation data is shown in the color associated with the relevant softening in the rest of the manuscript, while the experiments are black lines. The meaning of colors in the false color plots are shown in the two legends on the right hand side. All scale bars in h–j) are 10 mm.

**Figure 4:**
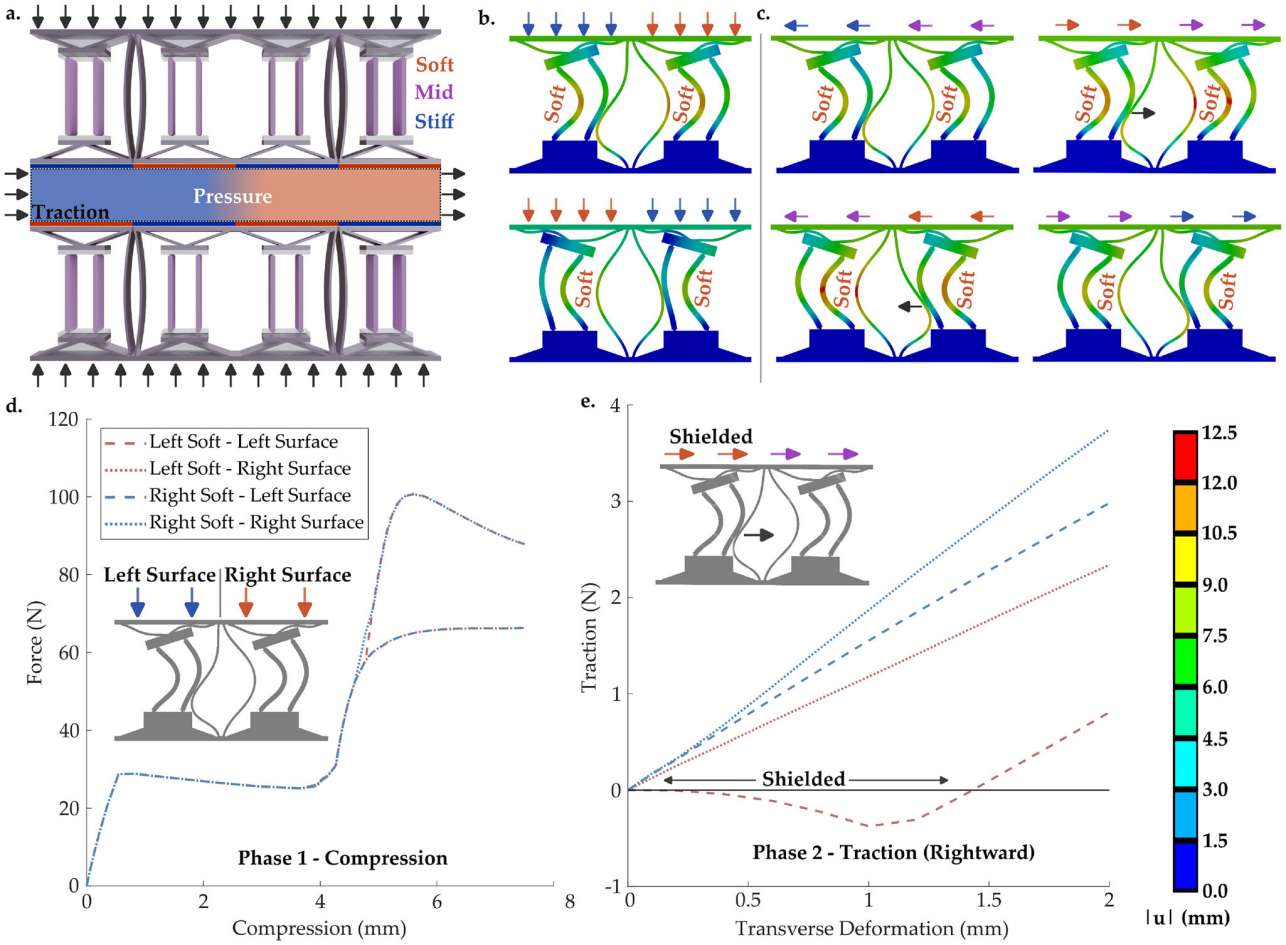
a) An arrangement that allows pressure and traction to be locally tuned. b) Deformation of the structure following selective beam softening ‐ showing resultant pressures on the compressed face. c) Local traction, following transverse deformation (that would be expected while gripping and picking objects, for example). d) Force vs. displacement relating to the various colored arrows in (b). e) Traction vs. displacement relating to the various colored arrows in (c) ‐ showing only rightward deformations for brevity. The relative meaning of arrow colors is described in a) (with soft meaning lower force or larger deformation, and vice versa), while the legend on the right hand side applies to the false color plots in b) and c).

**Figure 5:**
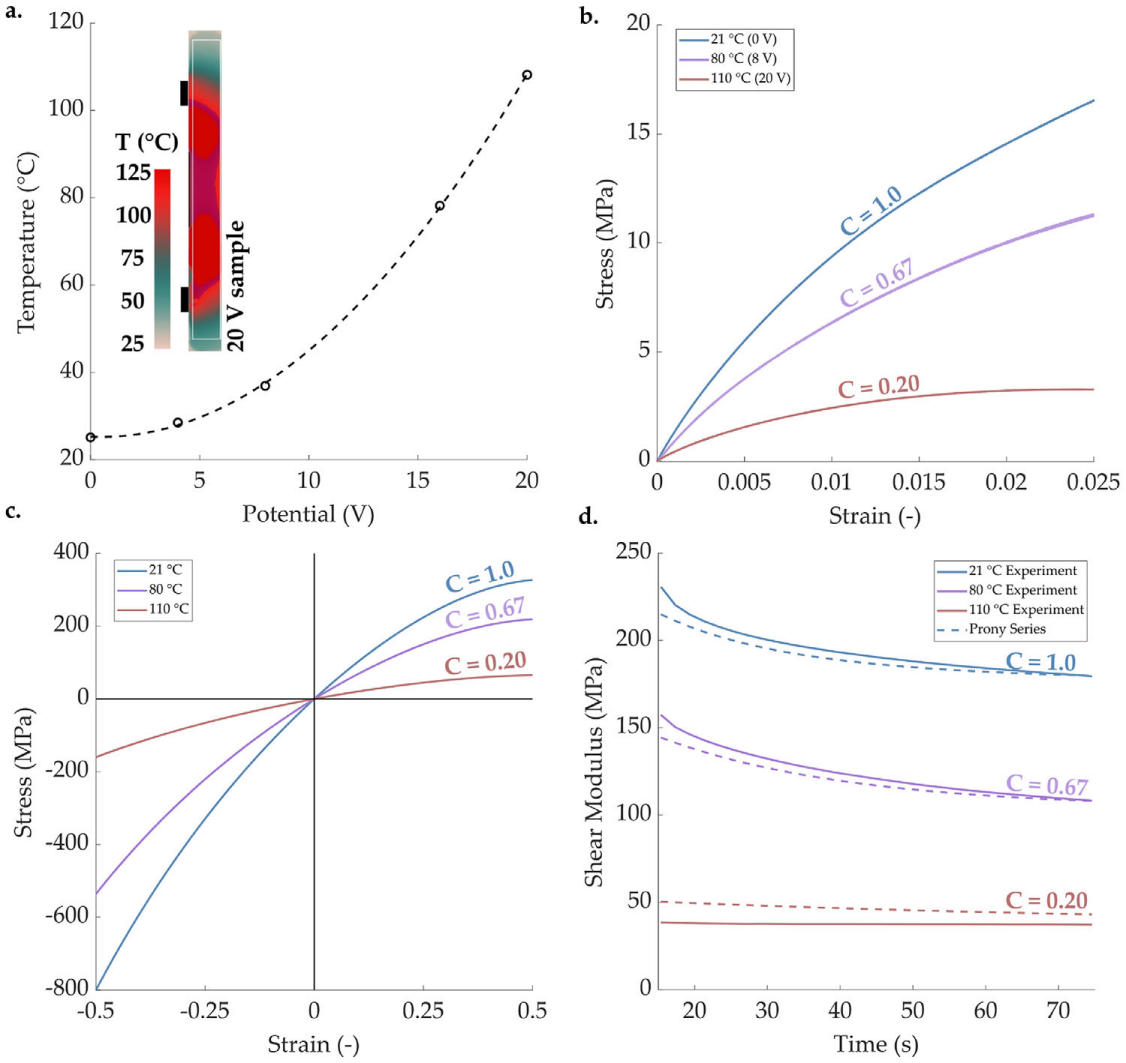
a) Temperature vs. applied voltage for the beam used throughout, including a false color plot from the thermal camera at an applied potential difference of 20 V, with circuit connection points shown as black rectangles. b) True stress vs. true strain data for tensile tests at all applied voltages (i.e., between 0 and 20 V). c) Neo–Hookean models extrapolated from data in (b). d) Prony series from stress‐relaxation data.

Herein, we have neither considered dynamic nor cyclic effects, given that polypropylene undergoes plastic deformation under moderate to high loads (see Supplementary Information, Section S2). Further work could explore a broader range of conductive polymers with more resistance to plastic deformation, such as TPU, to tune the concept for a wider range of potential applications.^[^ ^71^ ^]^ Similarly, further work could study concentrated loading, where deformation applied to different unit‐cells is non‐uniform, and expansions to 3D periodicity.

### 3.2 Programmable Nonreciprocity

Next, the conditions shown in (Figure 1f,g) are replicated, by loading single unit‐cells in simple shear. In the multi‐cell metamaterial, these unit‐cells have stiff connections, making these simplified tests equivalent to that of the fishbone‐like structure.^[^ ^74^ ^]^ While the symmetrical system shows a reciprocal response under simple shear (**Figure** **3**a), softening either of the two active beams causes a change in the force‐deformation relationship between *F*_*R*_ → *u*_*L*_ and *F*_*L*_ → *u*_*R*_ (Figure 3b,c). While the experiments were undertaken to a smaller applied deformation than simulations, to mitigate separation of bonded layers, relative stiffness and the nonreciprocal effects were still shown (Figure 3a–c). Namely, when the right hand beam is softened the system is ∼10% stiffer under deformation to the right‐hand side (*u*_*R*_/*F*_*L*_ > *u*_*L*_/*F*_*R*_), and vice versa. This nonreciprocity occurs because the moments on the top compliant mechanism change direction with the application of left or right loads, while those caused by tension do not. This asymmetry (and the change in stiffness of the active beams) causes either alignment, or misalignment of moments on the compliant mechanism, allowing it to either rotate and relax loading on the stiffer beam, or to remain horizontal, increasing the load applied to the stiffer beam. The plate rotation can be seen by comparing its vertical deformation on the left and right sides. The difference between vertical deformation on each side of the plate is larger when the forces applied to the set deformation are lower, suggesting more rotation (Figure 3e ‐ bottom, f ‐ top), and the horizontal plate deformation is also lower (Figure 3g ‐ top).

### 3.3 Pressure and Traction Control

This section showcases the potential for concepts similar to the considered metamaterial to be tuned for combined compressive and shear deformation (**Figure** **4**). Such a system may find applications in robotic grippers, and interfaces on custom medical devices. The considered metamaterial could form the interface covering stiff supportive or mechanical components (Figure 4a). Robotic grippers require fine motor control to carry out retrieval or maintenance on often fragile objects.^[^ ^17^, ^75^, ^76^ ^]^ These include agricultural goods farmed using robotic systems, human tissues during assisted surgery, or machinery and equipment that requires maintenance in extreme environments. Medical devices, such as supports and constraints used in surgeries, require modulation of contact between interchangeable surfaces.^[^ ^77^, ^78^ ^]^ The considered metamaterial is capable of switching between preset modes that locally modulate pressure on the surface of an object (Figure 4b,d), by switching individual unit‐cells between the modes analyzed in Figure 2. Further, these could then be used to tune traction, preventing damage (e.g., by wrinkling or tearing) of fragile surfaces (Figure 4c,e). Indeed, selected sections can be shielded from experiencing traction (Figure 4e). When the curved and active beams first undergo self‐contact (following compression), and are then moved apart by the applied simple shear deformation (Figure 4b,c), the stiffness of the system reduces, as in previous analyses of contact between curved beams.^[^ ^59^ ^]^ As such, the traction between the unit‐cell and the body applying the deformation remains close to zero, while increasing in other regions (Figure 4e). In order to realize this potential application, further work could apply the concept to a demonstrative set of grippers. Conductive polymers with greater reversibility/recovery than the polypropylene used here could be applied (such as newly developed ones based on TPU).^[^ ^71^ ^]^

## 4 Conclusion

An active mechanical metamaterial with smart, conductive polymers and a compliant mechanism was designed to switch between three compressive deformation modes: i) A soft mode, whereby beams buckle away from each other. ii) A stiff mode, whereby beams buckle toward each other. iii) A symmetrical mode, whereby buckling direction is random. Each of these modes also gives rise to nonreciprocity under shear loading. The system can be combined to form a surface capable of local pressure and traction modulation, with potential for use in soft robotic grippers and medical devices. This is the first active mechanical metamaterial leveraging these printable conductive polymers. The multifunctional system can be designed and printed or molded using readily available equipment and scalable processes, meaning there is a route from prototyping to scale‐up. This study paves the way for further work scaling these designs for specific applications, broadening to other conductive polymers, and causing modulated response changes by tuning variation between smart polymers (e.g., by using variable conductivity profiles).

## 5 Experimental Section

### Metamaterial Design and Fabrication

The considered metamaterial was initially designed to follow the basic relational requirements listed below, and then refined experimentally. First, the compressive and torsional stiffness of each active beam (while connected to the compliant mechanism) should change between being higher or lower than that of the compliant mechanism (Figure 1a). Second, the stiffness of the curved beam should be lower than that of the active beams (Figure 1a). The degrees of freedom used to modify these were the active beam dimensions (*d*_*b*_ and *t*_*b*_) and separation (*w*_*b*_), the thickness of the oblique ribs and curved beam (*t*, which were set to be equal), and the height of the compliant mechanism (*h*_*r*_, Figure 1a). The height of the unit‐cell (*h*) was set to 60 mm, and the width (*w*) was 44 mm. The overall depth was set to 10 mm, which was thick enough to prevent out‐of‐plane deformation. The internal feature dimensions were *t* = 1 mm, *R* = 145 mm, *h*_*r*_ = 5.9 mm, *w*_*r*_ = 34 mm, *t*_*p*_ = 5 mm, *w*_*p*_ = 20 mm, *h*_*b*_ = 33 mm, *w*_*b*_ = 10, *t*_*b*_ = 2.2, and *d*_*b*_ = 3.5 mm (Figure 1a). As the unit‐cell is surrounded by stiff plates that cause rigid boundaries and an effective Poisson's ratio of zero, there is a one‐to‐one mapping between constitutive properties of the unit‐cell and a multi‐cell (periodic) metamaterial under quasistatic loading conditions, which simplifies design and analysis (see Section S2, Supporting Information). For the system designed to demonstrate pressure and traction control under combined compression and shear deformation, the top plate of adjacent unit‐cells were connected with a 3 mm wide section of compliant materials, to allow relative movement.

The active beams consisted of polypropylene (Sabic CX03–81 Natural 00900, Supplied by Hardie Polymers), selected for its flexibility, doped with a 30% mass ratio of Carbon Black (C‐NERGY SUPER C65, supplied by PI‐KEM)^[^ ^69^ ^]^ (see Section S1, Supporting Information). The passive components were fabricated from polypropylene (Ultrafuse PP). Components were printed in the same orientation to ensure layer alignment, using 1.75 mm diameter filaments (Prusa i3 Mk3 FFF, with a 0.6 mm nozzle) with a rectilinear infill pattern of 100% and a layer height of 0.2 mm. The print bed temperature was 100°C while the nozzle temperature was 220°C. Polypropylene adhesive (Magigoo) and a 5‐mm brim were used to prevent samples from detaching from the print bed. The rig to apply simple shear and the inserts used to lock one layer of the compliant mechanism (Figure 3) were printed from polylactic acid (Ultrafuse PLA ‐ 1.75 mm), with nozzle temperature increased to 230°C, and similar settings to the other component. Additional plates to secure the active beams to were laser cut from 3 mm thick perspex. Components were typically bonded together with superglue (Loctite). To allow components to be replaced between compression tests, the perspex plates were bonded using double‐sided tape (RS PRO White). The active beams were replaced after each test.

### Smart Material Characterization

In this section, the conductive polymer was mechanically characterized (as previous work focused on the electrochemical response). A reduced order model between electrical inputs and mechanical properties was used to simplify the design process. First, the temperature of the printed, active beams was measured under different applied potential differences. Potential difference from a DC power supply (Thurlby, PL320 ‐ 30V/2A) was set to 4, 8, 16 or 20 V, while a thermal camera (FLIR A700 24° f/1.0 Prpromofessional S) with an IR‐lens (f = 18 mm (24°) f/1.0”) recorded temperature across the surface of the samples. Based on the current required to meet this 20 V potential in five of the active, additively manufactured beams, the resistance was 180 ± 10 Ω (mean ± standard deviation). This equates to a mean conductivity of 29.2 S/m, which was slightly lower than previously reported values for the filament (of ∼40 S/m), where repeatability between samples and charging cycles was also shown.^[^ ^69^ ^]^ Temperature settled after ∼30 seconds (see Section S4, Supporting Information), when a mean value across the whole surface was calculated (in FLIR Research Studio for Windows, **Figure** **5**a). The maximum temperature in the sample with a potential difference of 20 V was 130°C, noting that the melting point of polypropylene is usually ⩾165 °C.^[^ ^79^ ^]^ A 2nd order polynomial described the relationship between the sample temperature (*T*) and the applied potential difference (*V*) when tested at room temperature (*T*_*R*_).

(1) $$T=0.22V^{2}-0.21V+T_{R}$$

Mechanical tests were undertaken on a Tinius Olsen H50KS, equipped with a 1 kN load cell, with data recorded every millisecond (See Section S2, Supporting Information). Tests were filmed using a DSLR camera (Nikon D3200, resolution 1200 × 1080 p, frame rate 24 Hz), and a lens with 60 mm optical zoom (AF Micro Nikkor). The image plane of the camera was parallel to the face of the sample. Room temperature was ∼22°C, while relative humidity was between 40% and 60%.

To obtain elastic and viscoelastic material models, type‐iv ASTMD638‐14^[^ ^80^ ^]^ samples were tested in tension to 2.5% engineering strain, at an applied strain rate of 8.33 × 10^−4^ *s* ^−1^. Stress relaxation was undertaken by holding this strain for 70 s. The applied load was released at the same strain rate, to observe plasticity. These tests were repeated after connecting electric cables to either end of the gauge length (∼40 mm) of the samples, which were then connected to the power supply with potential differences set to 4, 8, 16, or 20 V (as per the temperature measurement tests). Samples were held at the applied potential difference for 30 s to allow temperature to settle before starting each test. True strain (ϵ) was obtained from sample gauge length (*l*), and the test device displacement (*u*), as (ϵ = *ln*(1 + *u*/*l*)). True stress (σ) was obtained from the test device's load cell and sample measurements (∼2.2 by ∼3.5 mm) taken before each test, using a vernier caliper (σ = *F*/(*A*(1 − ϵν)^2^)). Young's modulus (*E*) was obtained by fitting a straight line to the stress vs. strain data, up to a strain of 0.001. Poisson's ratio (ν) was assumed to be 0.49 (i.e., nearly incompressible).

Varying the applied potential difference between 0 and 20 V caused the beam to soften to approximately a fifth of its initial stiffness (Figure 5b). This gave an almost linear relationship between applied potential difference and relative stiffness (*C*), which could be defined as:

(2) C=1−0.04V

A Neo–Hookean model was also constructed (Figure 5c,^[^ ^81^ ^]^ route mean square error (RMSE) ⩽ 0.1):

(3) $$W=C_{1}\left(I_{1}-3\right)$$

whereby *I*_1_ is the first principle invariant, and *C*_1_ is half of the shear modulus (*G*):

(4) $$G=\frac{E}{2(1+\nu )}$$

The shear modulus at 21 °C was *G*_21_ = 460 MPa, based on the measured Young's modulus of 1,380 MPa (which is typical for polypropylene).^[^ ^79^ ^]^ The values at the other temperatures/applied potentials were considered directly proportional to the term *C* (i.e., Equation 2). While the onset of plasticity occurred at 10 MPa (see Section S2, Supporting Information), unloading was not considered in the simulations, meaning that this did not affect the analysis.

The visco‐elastic response of the material was represented using a seven term Prony series,^[^ ^82^, ^83^ ^]^ which was found to fit the experimental data (Figure 5d, RMSE ≈ 0.01):

(5) $$G_{t}=C\left(G_{21}+\sum_{i=1}^{7}\alpha_{i}e^{Ct_{i}}\right)$$

whereby *t*_*i*_ is relative time at one of the seven intervals required to construct the model, and the time‐dependent shear modulus term (α) is found by a least squared approximation for data recorded with no applied potential difference. The time data was expanded to convert to different temperatures, according to the principle of time vs. temperature superposition.^[^ ^83^ ^]^ All material properties recorded without an applied current (i.e., room temperature) are shown in **Table** **1**.

**Table 1:** Material properties and models at room temperature.

Material model	Coefficient value		
**Neo–Hookean (MPa)**			
*C*_1_	230		
**Prony Series**	α_*i*_ (*MPa*)	*t*_*i*_ (*s*)	
i=1	0.05676	39.74	
i=2	0.05664	14.49	
i=3	0.05675	39.74	
i=4	0.05665	13.59	
i=5	0.05666	14.49	
i=6	0.05665	13.59	
i=7	0.05666	13.62	
**Properties**			
ρ (*kg*/*m* ^3^)	*E* (*MPa*)	ν	*G*(*MPa*)
1000	1380	0.49	460

### Metamaterial Testing

Compression testing was undertaken using the same set up as the smart material characterization. Active beams were connected to the power supply (in parallel), in two groups (set to 0 or 20 V), to switch between the two required responses. A pre‐load of 5 N was applied to the samples, and then the specified potential difference was set 30 *s* before the test was started (allowing temperatures to settle). The 60 mm samples were compressed to 12 mm (i.e., 20% engineering strain), at a strain rate of 0.003 *s* ^−1^. Similarly, simple shear tests used to show the nonreciprocity were undertaken by fixing samples to a rigid rig that applied a transverse deformation of 3 mm at the same applied strain rate as the compression tests (see Section S2, Supporting Information). The force data from these shear tests was filtered with a low‐pass Butterworth filter (with cut‐off frequency of 0.1).

### Simulations

The simulations were undertaken as static structural analysis in ANSYS Mechanical, and reflected the experiments (although applied simple shear was increased to 7 mm, to amplify the causes of the nonreciprocity and explore them in more detail). Additional load cases, consisting of 7 mm of compression followed by 2 mm of transverse deformation, were also simulated (Figure 4f).

All bodies were imported into ANSYS Design Modeler as 2D surfaces, and topologies were shared. Quadrilateral mesh elements were applied with a minimum size of 0.5 mm, reduced to 0.25 mm over the thinner beams, providing at least three elements across each beam. A small section of material was removed where the beams met the compliant mechanism, to reflect the imperfect connection in the assembled structures. Frictional contacts that updated with each iteration were set between internal faces expected to come into contact, with a coefficient of friction of 0.2,^[^ ^79^ ^]^ a stabilization damping factor of 0.1, and a pinball radius of 1 mm. Large deformations, and non‐linear analysis were enabled in ANSYS, with a maximum step size of 10 s and a minimum of 0.02 s over the 100 s simulations. Constant energy stabilization with a dissipation ratio of 1× 10^−4^ and a stabilization force limit of 2 N was applied. See Section S3 (Supporting Information) for images showing meshing and boundary conditions.

The material models described in the materials characterization were applied to specific components. To reflect changes in material properties caused by changing the applied potential difference (and temperature), model parameters were adjusted according to Equation (2), Equation (3), and Equation (5). A Neo–Hookean material model was constructed for the polypropylene surround, based on typical material properties for polypropylene (*E* = 1,300 MPa).^[^ ^79^ ^]^ This was reduced to 900 MPa in the compliant mechanism ribs, where it was noticed that the print direction was approximately perpendicular.^[^ ^84^ ^]^ The stiff blocks were defined as structural steel in ANSYS Mechanical (E = 210 GPa, ρ = 7,850 *kg*/*m* ^3^).

## Conflict of Interest

The authors declare no conflict of interest.

## Supporting information

Supporting Information

Supporting Information

## Data Availability

The data that support the findings of this study are available in the supplementary material of this article.

## References

[1] B. Florijn , C. Coulais , M. Van Hecke , Phys. Rev. Lett. 2014, 113, 2. 10.1103/PhysRevLett.113.175503 25379923

[2] M. Brandenbourger , X. Locsin , E. Lerner , C. Coulais , Nat. Commun. 2019, 10, 1. 31601803 10.1038/s41467-019-12599-3 PMC6787071

[3] G. Librandi , E. Tubaldi , K. Bertoldi , Nat. Commun. 2021, 12, 1. 34103522 10.1038/s41467-021-23690-z PMC8187725

[4] M. Shaat , H. S. Park , J. Mech. Phys. Solids 2023, 171, 105163.

[5] H. Nassar , B. Yousefzadeh , R. Fleury , M. Ruzzene , A. Alù , C. Daraio , A. N. Norris , G. Huang , M. R. Haberman , Nat. Rev. Mater. 2020, 5, 667.

[6] J. Veenstra , C. Scheibner , M. Brandenbourger , J. Binysh , A. Souslov , V. Vitelli , C. Coulais , Nature 2025, 639. 10.1038/s41586-025-08646-3 40074911

[7] M. Y. Khalid , Z. U. Arif , A. Tariq , M. Hossain , R. Umer , M. Bodaghi , Mater. Des. 2024, 246, 113305.

[8] C. Coulais , D. Sounas , A. Alù , Nature 2017, 542, 461. 28192786 10.1038/nature21044

[9] C. Coulais , E. Teomy , K. de Reus , Y. Shokef , M. van Hecke , Nature 2016, 535, 529. 27466125 10.1038/nature18960

[10] G. W. Milton , The Theory of Composites, Cambridge University Press, Cambridge, UK, 2002.

[11] R. Craster , S. Guenneau , M. Kadic , M. Wegener , Rep. Prog. Phys. 2023, 86, 094501. 10.1088/1361-6633/ace069 37343550

[12] P. Yu , P. Zhang , Q. Ji , F. Yang , X. Tan , X. Chen , H. Tan , V. Laude , M. Kadic , Int. J. Solids Struct. 2024, 305, 113040.

[13] K. Bertoldi , V. Vitelli , J. Christensen , M. van Hecke , Nat. Rev. Mater. 2017, 2, 17066.

[14] L. Wang , J. A. Iglesias Martínez , K. K. Dudek , G. Ulliac , X. Niu , Y. Zou , B. Wang , V. Laude , M. Kadic , J. Appl. Mech. 2024, 91, 11.

[15] M. Kadic , G. W. Milton , M. van Hecke , M. Wegener , Nat. Rev. Phys. 2019, 1, 198.

[16] Y. Chen , M. Kadic , M. Wegener , Nat. Commun. 2021, 12, 3278. 34078904 10.1038/s41467-021-23574-2 PMC8172548

[17] Y. Wang , L. Li , D. Hofmann , J. E. Andrade , C. Daraio , Nature 2021, 596, 238. 34381233 10.1038/s41586-021-03698-7

[18] A. S. Gladman , E. A. Matsumoto , R. G. Nuzzo , L. Mahadevan , J. A. Lewis , Nat. Mater. 2016, 15, 413. 26808461 10.1038/nmat4544

[19] T. A. M. Hewage , K. L. Alderson , A. Alderson , F. Scarpa , Adv. Mater. 2016, 28, 10323. 27781310 10.1002/adma.201603959

[20] T. Ding , V. K. Valev , A. R. Salmon , C. J. Forman , S. K. Smoukov , O. A. Scherman , D. Frenkel , J. J. Baumberg , Proc. Natl. Acad. Sci. USA 2016, 113, 5503. 27140648 10.1073/pnas.1524209113 PMC4878511

[21] F. Huang , J. J. Baumberg , Nano Lett. 2010, 10, 1787. 20408552 10.1021/nl1004114

[22] V. K. Valev , J. J. Baumberg , C. Sibilia , T. Verbiest , Adv. Mater. 2013, 25, 2517. 23553650 10.1002/adma.201205178

[23] M. Su , Y. Song , Chem. Rev. 2022, 122, 5144. 34415152 10.1021/acs.chemrev.1c00303

[24] X. Yu , H. Cheng , M. Zhang , Y. Zhao , L. Qu , G. Shi , Nat. Rev. Mater. 2017, 2, 1.

[25] S. Garg , P. Singla , S. Kaur , R. D. Crapnell , C. E. Banks , S. Seyedin , M. Peeters , Small 2024, 2403320, 1. 10.1002/smll.202403320 39113348

[26] E. Bernalte , K. K. Augusto , R. D. Crapnell , H. G. Andrews , O. Fatibello‐Filho , C. E. Banks , RSC Appl. Interfaces 2024, 2, 439.

[27] M. Josselin , M. Castro , N. Di Cesare , F. Scarpa , A. Le Duigou , Adv. Mater. 2025, 2418656, 1. 10.1002/adma.202418656 PMC12016741 40072271

[28] K. K. Dudek , M. Kadic , C. Coulais , K. Bertoldi , Nat. Rev. Mater. 2025, 1.

[29] A. Rafsanjani , K. Bertoldi , A. R. Studart , Sci. Rob. 2019, 4, 29. 10.1126/scirobotics.aav7874 33137714

[30] J. S , N. K , V. M. T , Z. AA , Sci. Adv. 2020, 6, 616.

[31] R. Lakes , Science 1987, 235, 1038. 17782252 10.1126/science.235.4792.1038

[32] K. Wojciechowski , Phys. Lett. A 1989, 137, 60.

[33] J. N. Grima , K. E. Evans , J. Mater. Sci. Lett. 2000, 19, 1563.

[34] U. Larsen , O. Signund , S. Bouwsta , J. Microelectromech. Syst. 1997, 6, 99.

[35] J. N. Grima , A. Alderson , K. E. Evans , physica status solidi (b) 2005, 242, 561.

[36] R. Lakes , K. W. Wojciechowski , physica status solidi (b) 2008, 245, 545.

[37] J. C. L. Maxwell , Lond. Edinb. Philos. Mag. J. Sci. 1864, 27, 294.

[38] C. B. Churchill , D. W. Shahan , S. P. Smith , A. C. Keefe , G. P. McKnight , Sci. Adv. 2016, 2, 1. 10.1126/sciadv.1500778 PMC4788489 26989771

[39] K. E. Evans , A. Alderson , Adv. Mater. 2000, 12, 617.

[40] K. K. Dudek , O. Duncan , J. A. I. Martinez , M. Kadic , Adv. Sci. 2025. 10.1002/advs.202503088 PMC12407254 40538179

[41] J. A. Jackson , M. C. Messner , N. A. Dudukovic , W. L. Smith , L. Bekker , B. Moran , A. M. Golobic , A. J. Pascall , E. B. Duoss , K. J. Loh , C. M. Spadaccini , Sci. Adv. 2018, 4, eaau6419. 30539147 10.1126/sciadv.aau6419 PMC6286172

[42] J. Cui , T.‐Y. Huang , Z. Luo , P. Testa , H. Gu , X.‐Z. Chen , B. J. Nelson , L. J. Heyderman , Nature 2019, 575, 164. 31695212 10.1038/s41586-019-1713-2

[43] K. Dudek , J. I. Martínez , L. Hirsinger , M. Kadic , M. Devel , J. Sound Vib. 2025, 595, 118784.

[44] K. K. Dudek , J. A. Iglesias Martínez , G. Ulliac , L. Hirsinger , L. Wang , V. Laude , M. Kadic , Adv. Mater. 2023, 35, 2210993. 10.1002/adma.202210993 36863399

[45] J. Sim , S. Wu , S. Hwang , L. Lu , R. R. Zhao , Adv. Funct. Mater. 2020, 35, 2422325.

[46] H. Zhang , X. Guo , J. Wu , D. Fang , Y. Zhang , Sci. Adv. 2018, 4, eaar8535. 29888326 10.1126/sciadv.aar8535 PMC5993477

[47] Y. Tang , Y. Li , Y. Hong , S. Yang , J. Yin , Proc. Natl. Acad. Sci. USA 2019, 116, 26407. 31843912 10.1073/pnas.1906435116 PMC6936366

[48] X. Ni , X. Guo , J. Li , Y. Huang , Y. Zhang , J. A. Rogers , Adv. Mater. 2019, 31, 1905405. 10.1002/adma.201905405 31595583

[49] X. Guo , X. Ni , J. Li , H. Zhang , F. Zhang , H. Yu , J. Wu , Y. Bai , H. Lei , Y. Huang , J. A. Rogers , Y. Zhang , Adv. Mater. 2021, 33, 2004919. 10.1002/adma.202004919 33289278

[50] Y.‐C. Cheng , H.‐C. Lu , X. Lee , H. Zeng , A. Priimagi , Adv. Mater. 2020, 32, 1906233. 10.1002/adma.201906233 31834665

[51] Q. Zhang , X. Kuang , S. Weng , Z. Zhao , H. Chen , D. Fang , H. J. Qi , ACS Appl. Mater. Interfaces 2020, 12, 17979. 32196302 10.1021/acsami.0c02038

[52] L. A. E. Müller , T. Zimmermann , G. Nyström , I. Burgert , G. Siqueira , Adv. Funct. Mater. 2020, 30, 2002914.

[53] C. Scheibner , A. Souslov , D. Banerjee , P. Surówka , W. T. Irvine , V. Vitelli , Nat. Phys. 2020, 16, 475.

[54] Q. Ji , J. Moughames , X. Chen , G. Fang , J. J. Huaroto , V. Laude , J. A. I. Martínez , G. Ulliac , C. Clévy , P. Lutz , K. Rabenorosoa , V. Guelpa , A. Spangenberg , J. Liang , A. Mosset , M. Kadic , Commun. Mater. 2021, 2, 0.

[55] K. Zhang , J. Ji , X. Kang , X. Guo , Extreme Mech. Lett. 2024, 72, 102241.

[56] X. Ni , H. Luan , J. T. Kim , S. I. Rogge , Y. Bai , J. W. Kwak , S. Liu , D. S. Yang , S. Li , S. Li , Z. Li , Y. Zhang , C. Wu , X. Ni , Y. Huang , H. Wang , J. A. Rogers , Nat. Commun. 2022, 13, 1. 36151092 10.1038/s41467-022-31092-y PMC9508113

[57] K. Zhang , J. Ji , X. Kang , X. Guo , Extreme Mech. Lett. 2024, 72, 102241.

[58] C. El Helou , P. R. Buskohl , C. E. Tabor , R. L. Harne , Nat. Commun. 2021, 12, 1. 33712597 10.1038/s41467-021-21920-y PMC7954845

[59] J. S. Chen , L. C. Wang , Int. J. Solids Struct. 2020, 199, 181.

[60] P. Jiang , S. Zhang , H. Yang , Y. Li , ACS Appl. Mater. Interfaces 2023, 15, 43102. 37561821 10.1021/acsami.3c06463

[61] L. J. Kwakernaak , A. Guerra , D. P. Holmes , M. van Hecke , Extreme Mech. Lett. 2024, 69, 102160.

[62] Z. Zhang , S. Pusateri , B. Xie , N. Hu , Extreme Mech. Lett. 2020, 37, 100732.

[63] T. Chen , M. Pauly , P. M. Reis , Nature 2021, 589, 386. 33473228 10.1038/s41586-020-03123-5

[64] L. J. Kwakernaak , M. Van Hecke , Phys. Rev. Lett. 2023, 130, 1. 10.1103/PhysRevLett.130.268204 37450791

[65] D. Haid , Ph.D. thesis, Sheffield Hallam University, 2020.

[66] R. D. Crapnell , E. Bernalte , A. G. M. Ferrari , M. J. Whittingham , R. J. Williams , N. J. Hurst , C. E. Banks , ACS Meas. Sci. Au. 2022, 2, 167. 36785725 10.1021/acsmeasuresciau.1c00046 PMC9838814

[67] R. D. Crapnell , C. Kalinke , L. R. G. Silva , J. S. Stefano , R. J. Williams , R. A. Abarza Munoz , J. A. Bonacin , B. C. Janegitz , C. E. Banks , Mater. Today 2023, 71, 73.

[68] H. Yuk , B. Lu , S. Lin , K. Qu , J. Xu , J. Luo , X. Zhao , Nat. Commun. 2020, 11, 4. 32231216 10.1038/s41467-020-15316-7 PMC7105462

[69] D. L. Ramos , R. D. Crapnell , R. Asra , E. Bernalte , A. Oliveira , R. A. Mu noz , E. M. Richter , A. M. Jones , C. E. Banks , ACS Appl. Mater. Interfaces 2024, 16, 56006. 39358698 10.1021/acsami.4c12967 PMC11492246

[70] S. Nouseen , K. Ghosh , M. Pumera , Electrochem. Commun. 2024, 160, 107652.

[71] A. C. Oliveira , E. Bernalte , R. D. Crapnell , M. J. Whittingham , R. A. Mu noz , C. E. Banks , Applied Mater. Today 2025, 42.

[72] R. D. Crapnell , A. Tridente , C. E. Banks , N. C. Dempsey‐Hibbert , Sensors 2021, 21, 1. 10.3390/s21030879 PMC7865822 33525567

[73] B. Ferreira , R. D. Crapnell , E. Bernalte , T. R. Paix ao , C. E. Banks , Electrochimica Acta 2025, 515.

[74] J. Wang , Q. Ji , M. Kadic , C. Wang , Thin Walled Struct. 2024, 205, 112373.

[75] D. J. Levine , K. T. Turner , J. H. Pikul , Adv. Mater. 2021, 33, 1. 10.1002/adma.202007952 34245062

[76] R. Wu , P. C. E. Roberts , S. Lyu , F. Zheng , C. Soutis , C. Diver , D. Zhou , L. Li , Z. Deng , Adv. Funct. Mater. 2020, 2008252.

[77] J. U. Surjadi , L. Gao , H. Du , X. Li , X. Xiong , N. X. Fang , Y. Lu , Advanced Engineering Materials 2019, 21, 1.

[78] P. Suhas , J. D. Quadros , Y. I. Mogul , M. Mohin , A. Aabid , M. Baig , O. S. Ahmed , Materials Advances 2024, 6, 887.

[79] N. P. Cheremisinoff , in Condensed Encyclopedia of Polymer Engineering Terms , vol 1, Butterworth‐Heinemann, Oxford, 2001, pp. 200–255.

[80] American Society for Testing and Materials , ASTM International 2016, 82, 1.

[81] S. E. Bechtel , R. L. Lowe , in Fundamentals of Continuum Mechanics (Eds. R. L. L. Stephen , E. Bechtel ), Academic Press, Cambridge, US 2015 pp. 181–195.

[82] S. W. Park , R. A. Schapery , Int. J. Solids Struct. 1999, 36, 1653.

[83] J. D. Ferry , Viscoelastic Properties of Polymers , 3rd edition, 1980.

[84] T. D. Ngo , A. Kashani , G. Imbalzano , K. T. Nguyen , D. Hui , Composites, Part B 2018, 143, 172.

