# Supplementary material for: Electro‐Thermally Controlled Active Mechanical Metamaterials with Programmable Stiffness and Nonreciprocity
Source: Adv Sci (Weinh). 2025 Aug 28;12(43):e11669. doi: 10.1002/advs.202511669 (PMC12631830; doi:10.1002/advs.202511669)
Supplement: Supplementary file 1 — Supporting Information [file ADVS-12-e11669-s001.pdf]

Supplementary Information for Electro-Thermally Controlled Active Mechanical Metamaterials with Programmable Stiffness and Nonreciprocity

*Jai Dunne Robert D. Crapnell Krzysztof K. Dudek Tom Allen Craig E. Banks Olly Duncan**

J. Dunne, Dr R. D. Crapnell, Dr T. Allen, Prof C. E. Banks, Dr O. Duncan
Faculty of Science and Engineering, Dalton Building, Manchester Metropolitan University, Manchester, M1 5GD, UK
Email Address: O.Duncan@mmu.ac.uk
Prof K. K. Dudek
Institute of Physics, University of Zielona Gora, ul. Szafrana 4a, Zielona Gora, 65-069 Poland

S1 - Conductive Polymer Preparation

The polymer used for the active beams was made by doping the polypropylene (Sabic CX03–81 Natural 00900, Supplied by Hardie Polymers), with Carbon Black (C-NERGY SUPER C65, supplied by PI-KEM) [1]. This formulation was developed for use as a flexible electrochemical sensor [1]. The polypropylene was mixed with a 30% mass ratio of the Carbon Black at 210°C. The filament was then extruded (Filabot, EX2) to a diameter of 1.75 mm, making it compatible with the printer used to make the beams (Prusa i3 Mk3S+, with a 0.6 mm nozzle).

S2 - Mechanical Testing

All tests used a similar set up, as shown in Figure S1. The tensile load/unload cycles undertaken for the smart polymer initially showed a linear elastic response, followed by a lower gradient as it underwent plastic deformation (Figure S2). Plasticity is evidenced by the negative stress upon return to the reference position. The onset of plasticity was at approximately 10 MPa irrespective of the polymer temperate. As such, applying charge to the polymer reduced plasticity over a specific strain range.

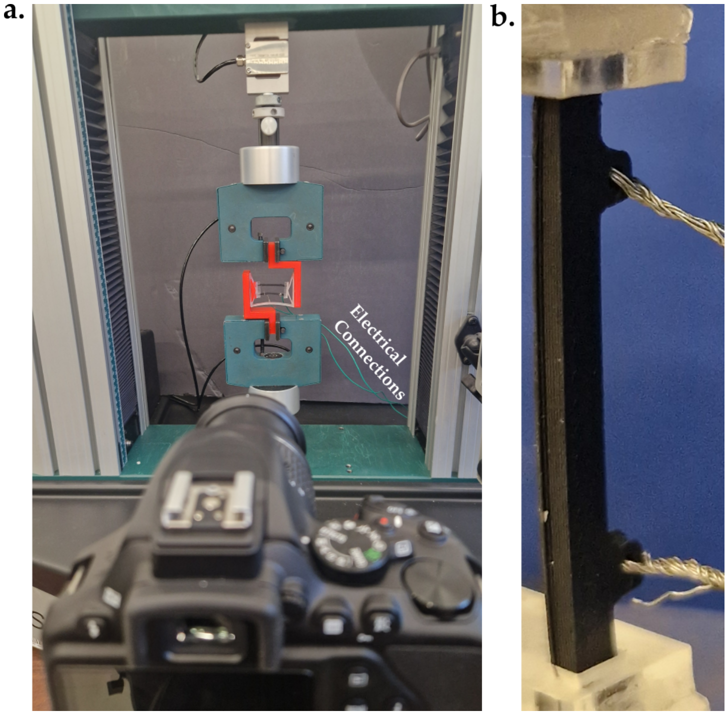

Figure S1: a) Testing set up, showing camera, uniaxial test device, sample (ready to be tested in shear), and electronic connections. b) Image showing the loops used to connect the circuit to the active beams.

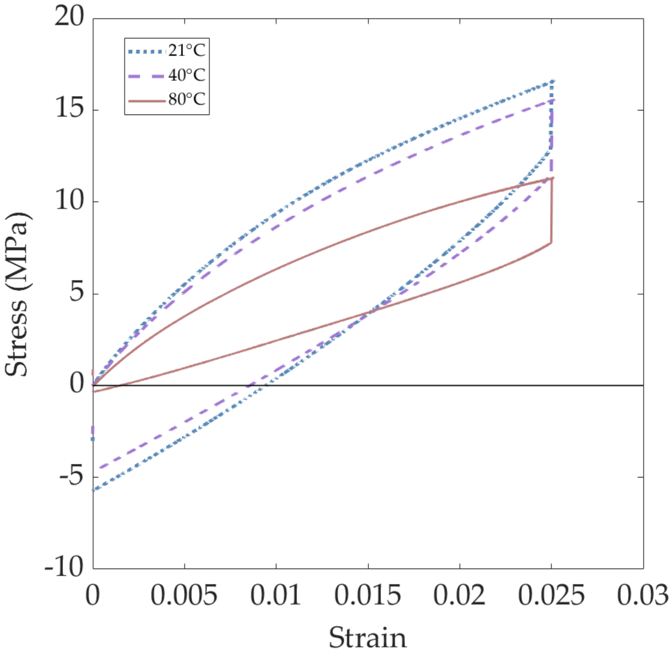

Figure S2: Engineering stress vs. strain covering the whole load, hold and unload cycle (21°C was room temperature).

Tests were also undertaken for single unit-cells, following methods described in the manuscript. An additional unload phase was included (at the same rate as applied compression). While scaling from a single unit-cell to a four by one array, emergent effects (from interactions between unit-cells) were generally not observed for either the blank passive elements, or the active metamaterial (Figure S3). This is because boundaries between cells had rigid couplings, that did not rotate or transmit traction between unit-cells in uniaxial tests. There was, however, an observable difference for the active system, when scaling from a unit-cell to repeated unit-cells. Namely, the multi-cell metamaterial shows smoother stiffness transitions between compressive deformation regions (i.e. buckling and self-contact). The effect of manufacturing imperfections that influence buckling, and internal contacts, differ between unit-cells, causing sharper transitions when testing one unit-cell, and more gradual ones when testing the multi-cell metamaterial. Considering the unloading phase, while some recovery was evident, the active beams remain partially deformed after buckling (Figure S3). Finally, considering the system without any active beams, during the compression, the stiffness was five times lower than that of the whole system, because the curved beam's compressive stiffness was half that of the active elements (Figure S3). Under simple shear, the system without active beams was substantially softer (Figure S4).

S3 - Finite Element Simulations

The applied mesh, and boundary conditions, are shown in (Figure S5). As the stiffness of either of the beams is gradually softened, there is little change in stiffness before the point of self contact (Figure S6). This is because the active beams remain stiffer than the compliant mechanism, meaning the compliant mechanism compresses before the active beams compress. The deformation here is similar to that shown for the symmetrical mode in Figures 1d and 2b of the manuscript. For simulations representing applied potentials of up to 20V, there was no change in buckling mode relating to the softening of either beam. These lower softening values do, however, cause a change in stiffness after self-contact occurs. When the value for stiffness of the active beam used in the manuscript is reached ($C = 0.2$, at a potential difference of 20 V), the switch in buckling mode occurs. As the stiffness of either beam is reduced further ($C = 0.17$), the active component of the structure becomes increasingly soft, and self-contact has minimal effect on stiffness. As the active beam is made even softer, and then removed, stiffness is substantially lower and buckling is prevented; the remaining beam is always in flexure.

S4 - Thermal Characterization

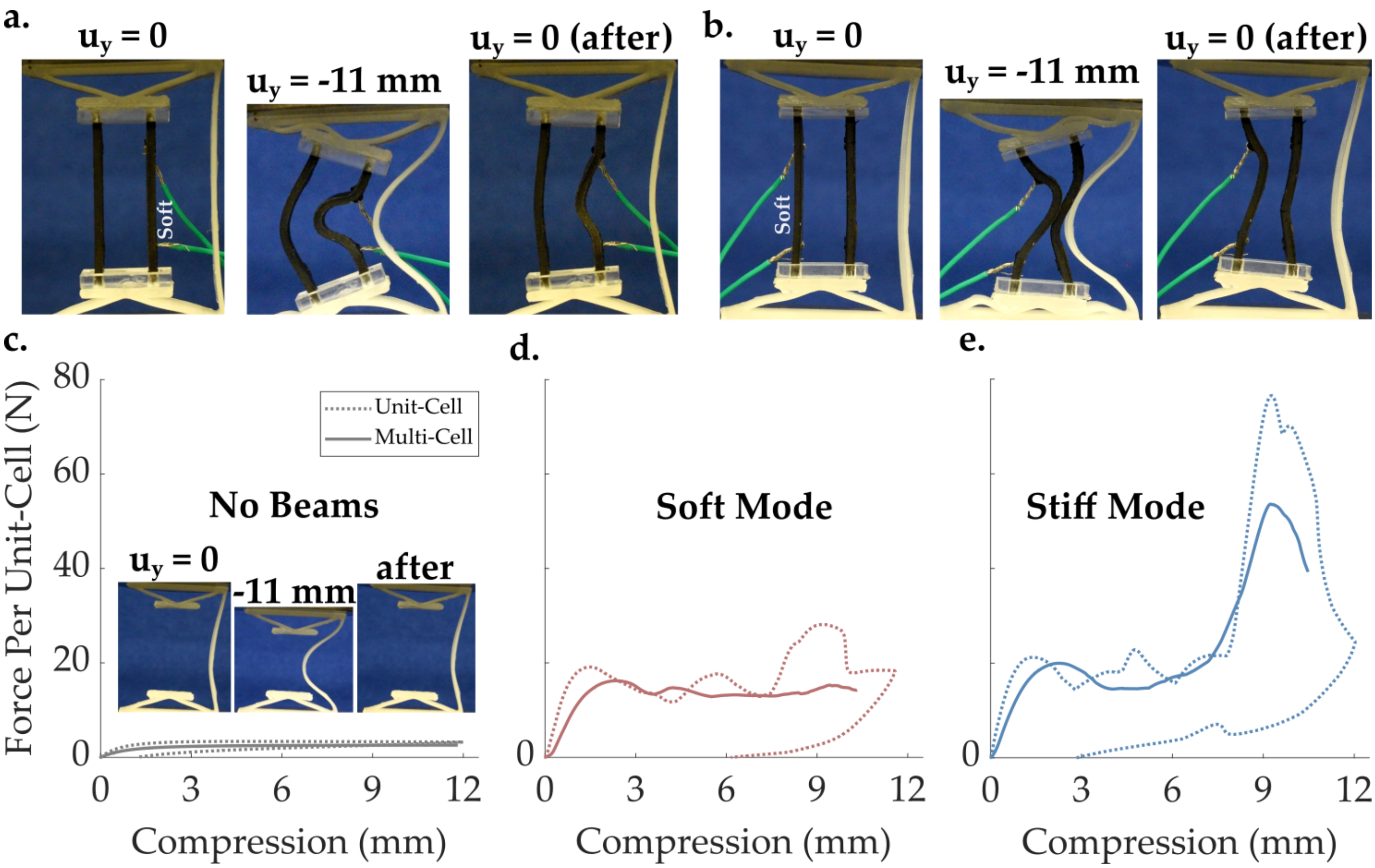

Figure S3: Experimental data for the single unit-cell, and multi-cell metamaterial. a) Images before, after, and at maximum compression, from videos of load-unload tests, with the beam closest to the passive curved element being softened. b) Images with the beam closest to the passive curved element being softened. c) to d) Force vs. displacement data for single and multiple unit-cells for c) Blank unit-cells without beams, d) the soft mode, and e) the stiff mode. The same y-axis is used for c) to e).

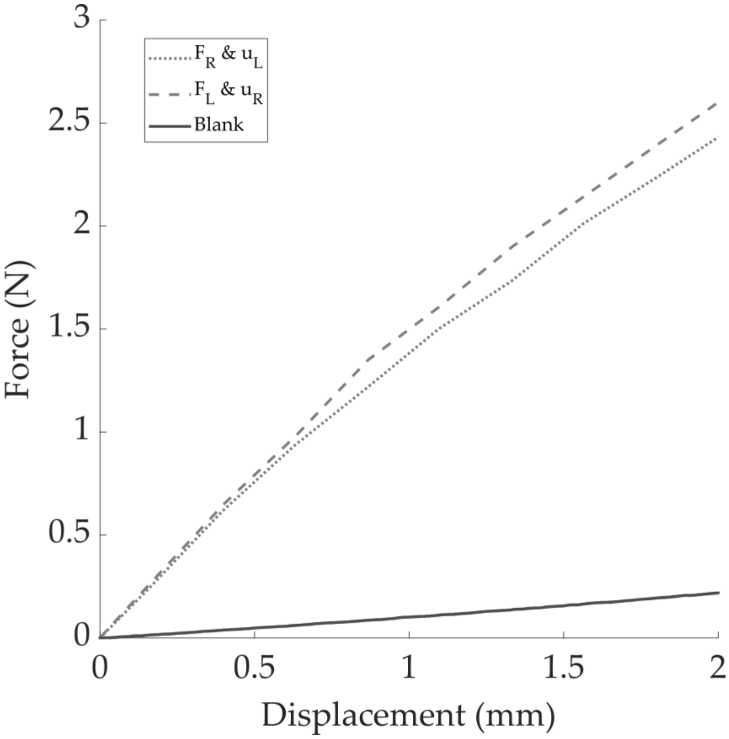

Figure S4: Experimental data from simple shear tests, that also include a blank unit-cell without the active elements.

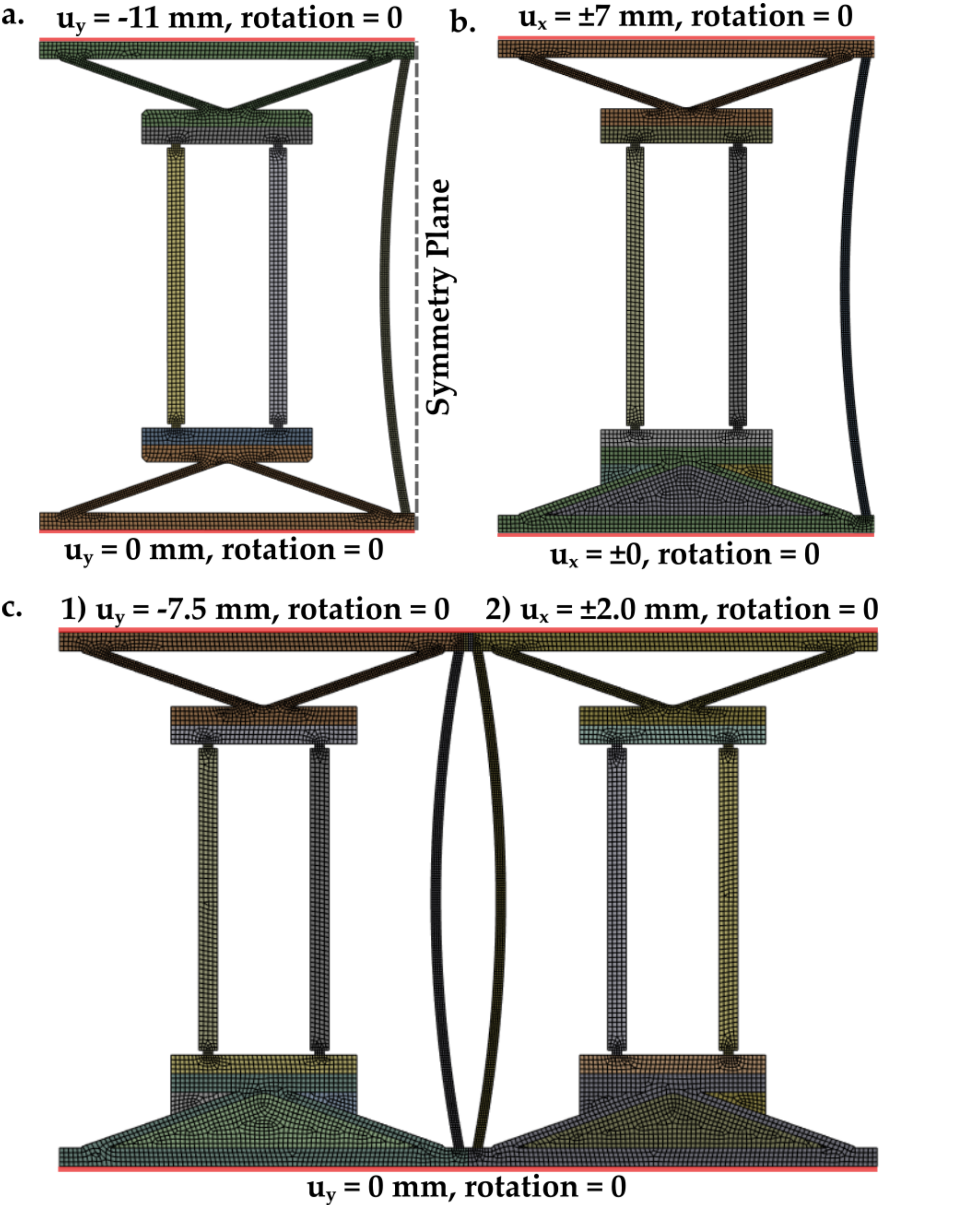

Figure S5: Mesh and applied boundary conditions for a) compression testing, and b) simple shear tests, used to show non-reciprocity. c) For the demonstrative coupled deformations - showing the 2-stages of deformation.

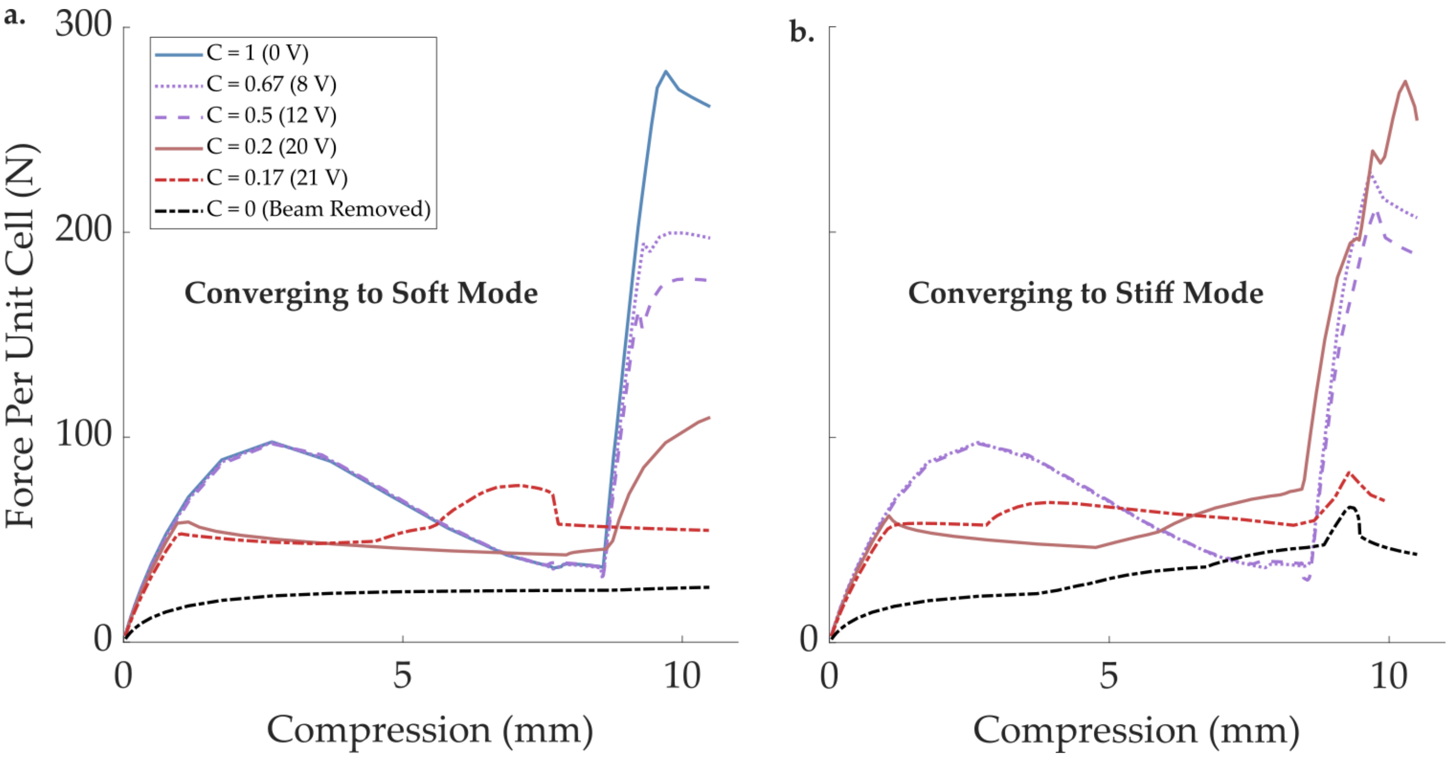

Figure S6: Simulations varying the constant C (in manuscript equations 2 to 5), that modulates stiffness of a) the active beam closer to the curved beam, causing convergence to the soft mode, and b) the active beam further from the curved beam, causing convergence to the stiff mode. The brackets in the legend in (a) show the potential differences required to reach the specified value of C (from equation 2 in the manuscript). $C = 1$ in a) is not repeated in b), as this configuration is symmetrical, meaning that both would be equal.

After the charge was first applied, the surface temperature was generally higher near the nodes where the circuit was connected (Figure S7a). Over time, the temperature became more even over the whole sample. For the 20 V sample, equilibrium was reached (and the temperature stabilized) after approximately 30 seconds - with the resistive heating being dissipated into the surroundings (Figure S7b). For the samples with a lower applied voltage (causing a smaller thermal gradient), this took closer to 40 seconds.

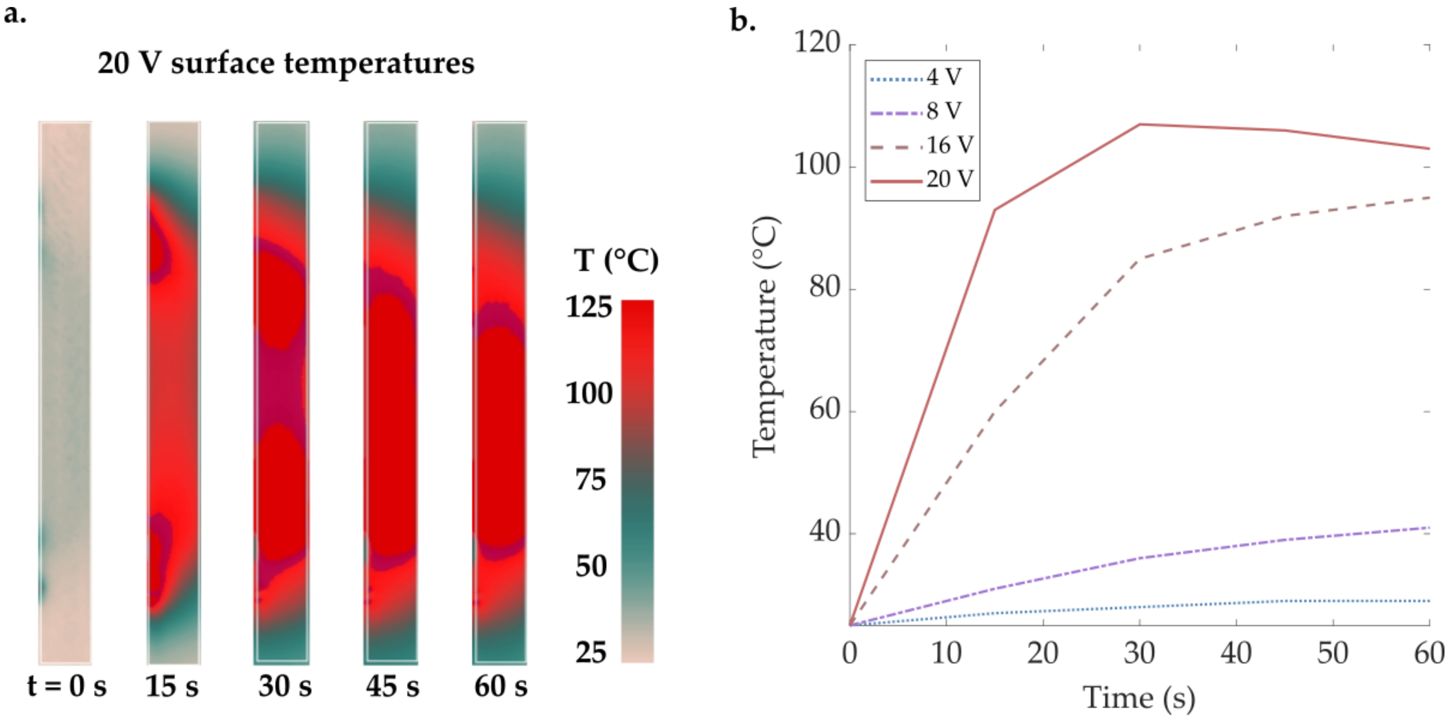

Figure S7: a) False color plots of surface temperature at 15 second time intervals, with an applied potential of 20 V (legend on right hand side). b) Mean surface temperature vs. time for the various potential differences, applied at t = 0 s.

References

[1] David L.O. Ramos et al. "Conductive Polypropylene Additive Manufacturing Feedstock: Application to Aqueous Electroanalysis and Unlocking Nonaqueous Electrochemistry and Electrosynthesis". In: *ACS Applied Materials and Interfaces* 16 (2024), pp. 56006–560018. ISSN: 19448252.
