# Supplementary figures and images for: Electro‐Thermally Controlled Active Mechanical Metamaterials with Programmable Stiffness and Nonreciprocity
Source: Adv Sci (Weinh). 2025 Aug 28;12(43):e11669. doi: 10.1002/advs.202511669 (PMC12631830; doi:10.1002/advs.202511669)

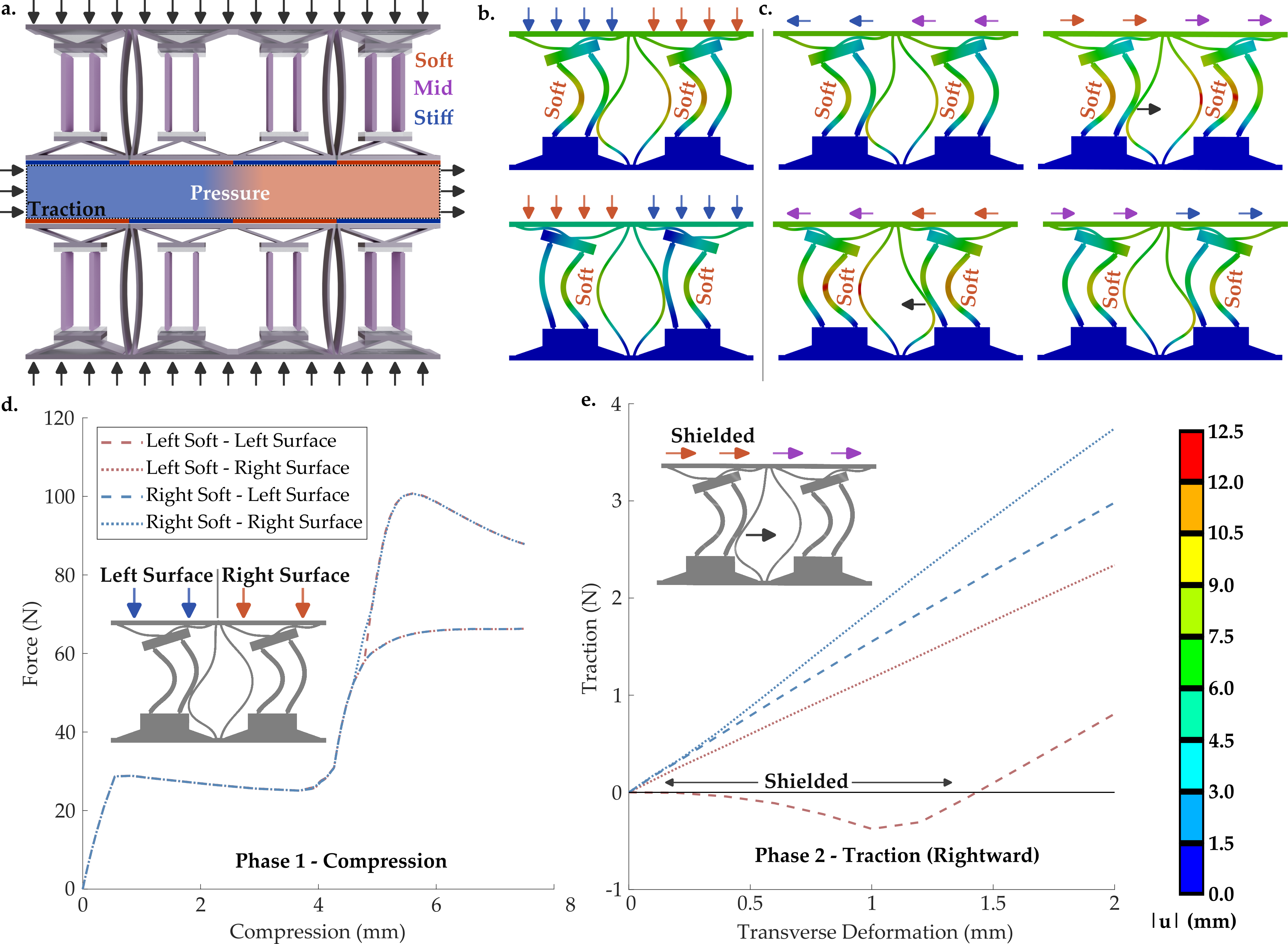

Supplement: Supplementary file 2 — Supporting Information [file ADVS-12-e11669-s002.zip › Application.png]

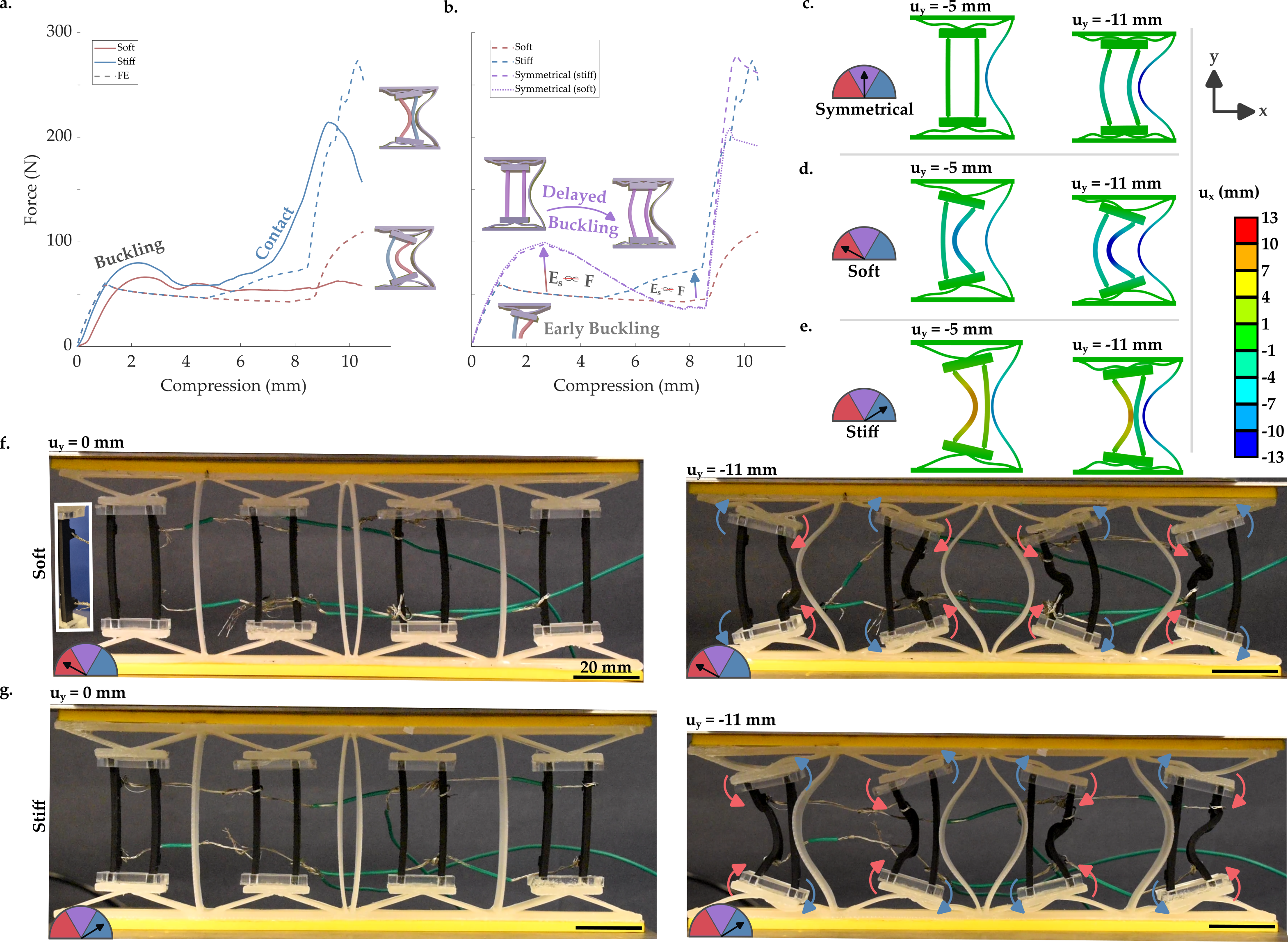

Supplement: Supplementary file 2 — Supporting Information [file ADVS-12-e11669-s002.zip › Compression.png]

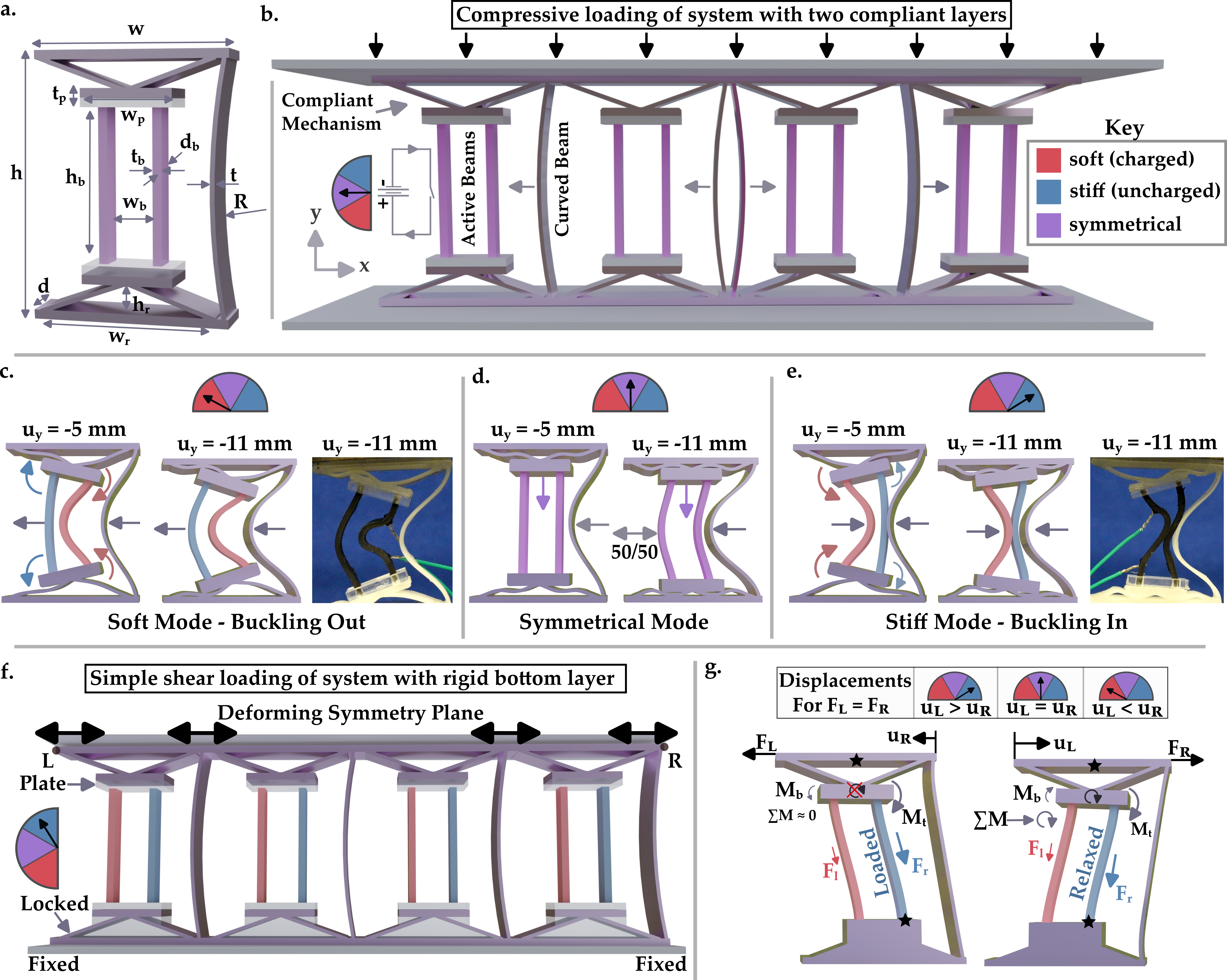

Supplement: Supplementary file 2 — Supporting Information [file ADVS-12-e11669-s002.zip › Concept.png]

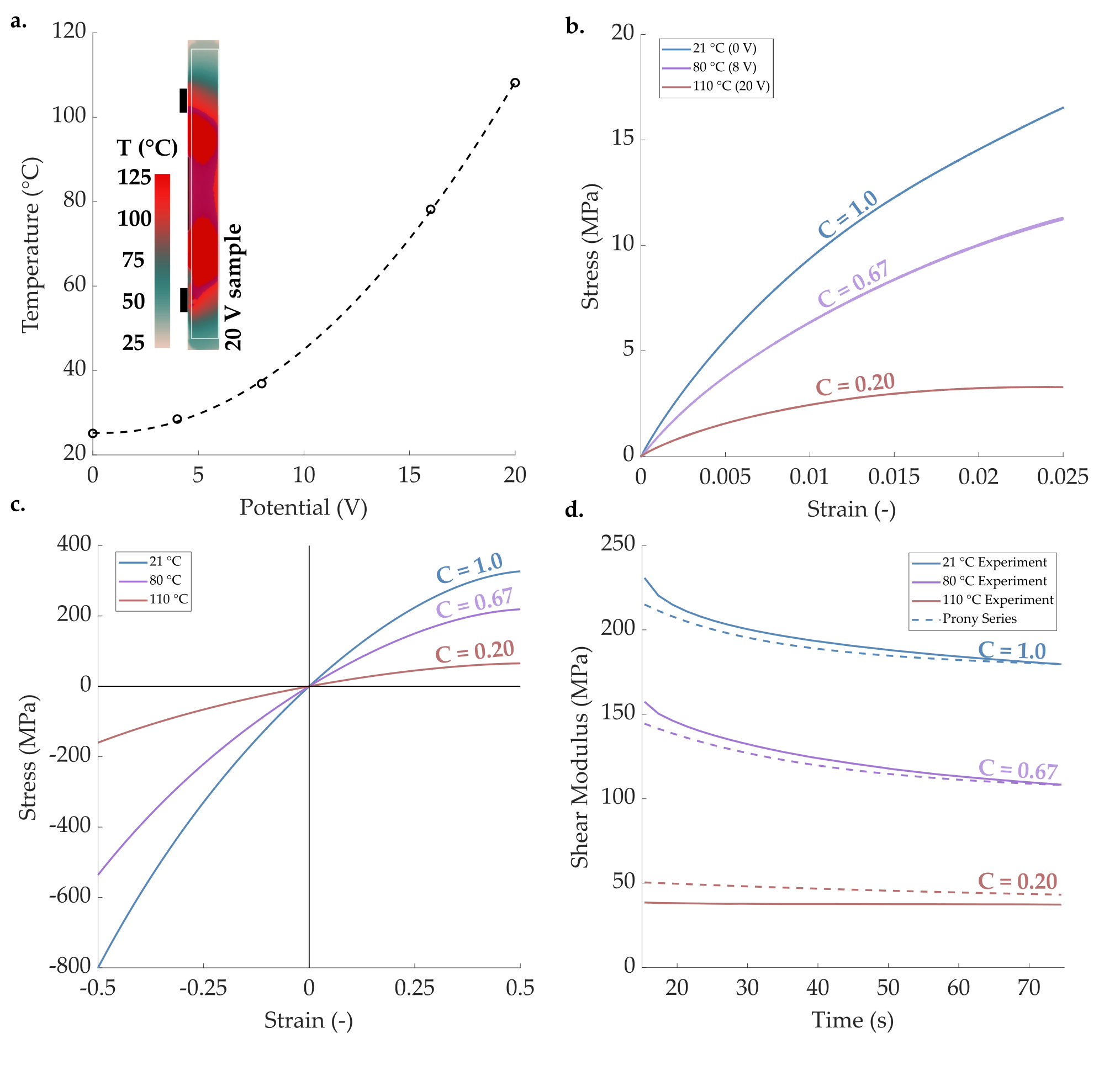

Supplement: Supplementary file 2 — Supporting Information [file ADVS-12-e11669-s002.zip › MaterialProps.png]

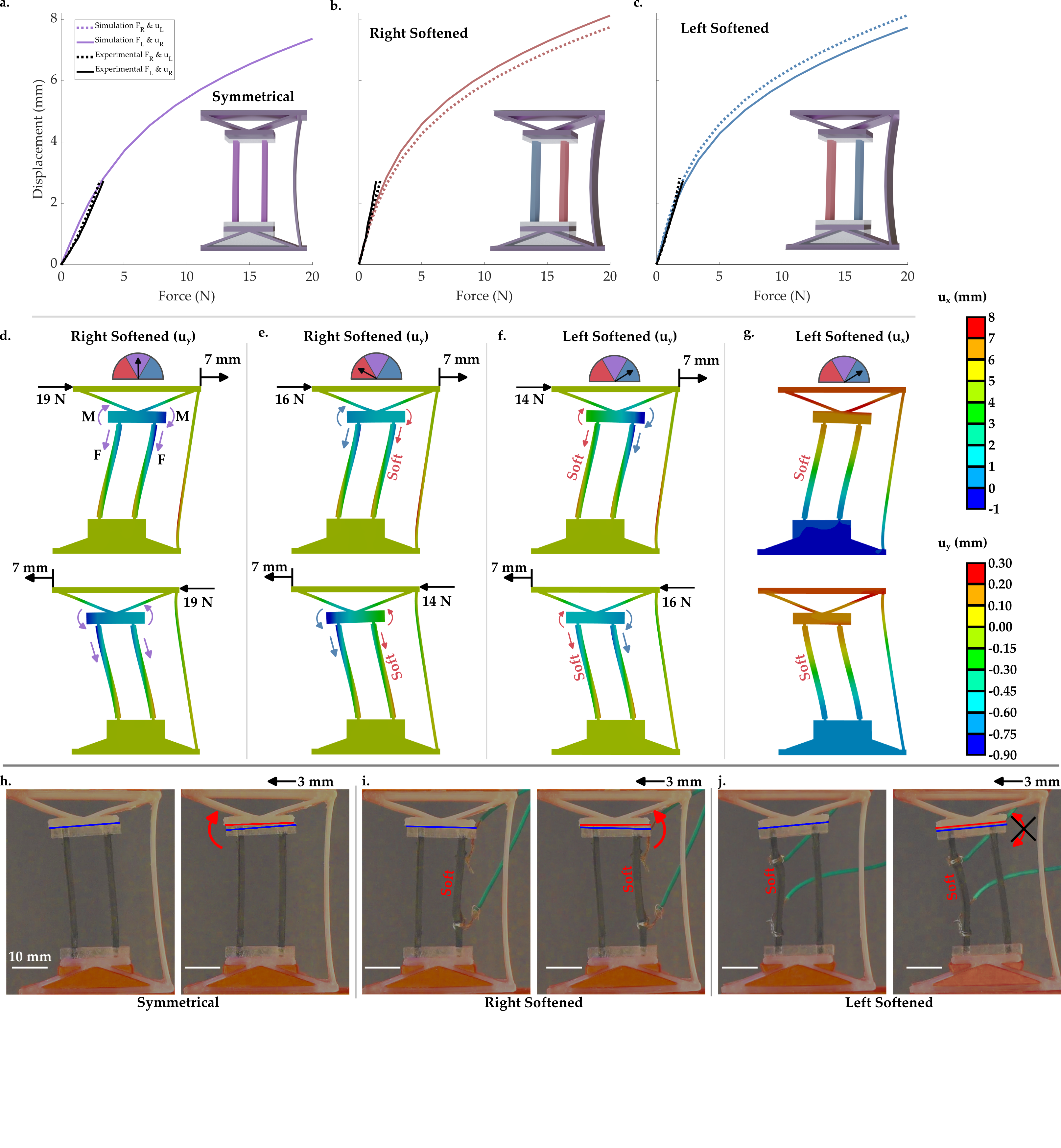

Supplement: Supplementary file 2 — Supporting Information [file ADVS-12-e11669-s002.zip › NR.png]
